# Nanotherapeutic Macrophage‐Neuro Reprogramming Through Immunometabolic Crosstalk Mitigates Sepsis‐Induced Lung Injury and Neurologic Damage

**DOI:** 10.1002/advs.202520665

**Published:** 2026-02-16

**Authors:** Wenhui Wang, Yongrui Hai, Xintong Lu, Ye Chen, Bingjie Zhang, Renming Fan, Jiarui Dou, Jiaxin Yan, Shuo Fu, Wen Zhang, Junke Song, Gaofei Wei

**Affiliations:** ^1^ Laboratory of Cellular Metabolism and Precision Therapeutics School of Life Science and Technology Northwestern Polytechnical University Xi'an P. R. China; ^2^ Research & Development Institute of Northwestern Polytechnical University in Shenzhen Shenzhen P. R. China; ^3^ Institute of Materia Medica Chinese Academy of Medical Sciences & Peking Union Medical College Beijing P. R. China

**Keywords:** lung injury, macrophage‐neuro reprogramming, nanotherapy, neuronal repair, sepsis

## Abstract

Sepsis remains a leading cause of mortality in intensive care units, with its associated organ dysfunction primarily driven by uncontrolled inflammation and neuroimmune dysregulation. Among affected organs, the lung is particularly vulnerable, with injury involving both immune‐mediated tissue damage and inflammation‐induced neuronal impairment. Yet, whether coordinated targeting of immune and neural compartments can achieve synergistic and durable therapeutic benefits remains unknown. Here, we report a rationally engineered, dual‐functional, enzyme‐responsive nanoplatform (SJNPs) that co‐delivers the glutamate production inhibitor JHU083 and the neuroprotective spermine (Spm) to reprogram macrophage‐neuron immunometabolic interactions. SJNPs suppressed pro‐inflammatory, M1‐associated macrophage activation while promoting M2 polarization, which in turn drove robust secretion of the neurotrophic factor nerve growth factor (NGF) and preserved pulmonary neuronal integrity. Mechanistically, inhibition of glutamate metabolism reprogrammed macrophage polarization and activated NGF‐mediated neurotrophic signaling, establishing NGF as a key mediator linking immune modulation to neural protection. In murine sepsis models, SJNPs attenuated systemic cytokine storms, mitigated alveolar damage, alleviated neurological injury, and improved survival. This study identifies macrophage‐neuron immunometabolic crosstalk as a previously underexplored therapeutic target for septic lung injury characterized by neuronal damage, and establishes metabolic reprogramming of macrophages as a promising strategy for integrated immunomodulatory and neuroprotective therapy in sepsis.

## Graphical abstract

SJNPs co‐deliver JHU083 and spermine to reprogram macrophage–neuron immunometabolic crosstalk in sepsis. By suppressing pro‐inflammatory M1 polarization and promoting NGF‐mediated neurotrophic signaling, SJNPs preserve pulmonary neuronal integrity, alleviate lung injury, and improve survival in murine sepsis models.

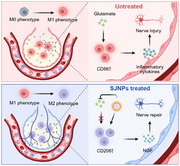

## 1 Introduction

Sepsis is a life‐threatening syndrome triggered by infection and characterized by dysregulated host responses leading to multi‐organ dysfunction and high mortality rates [1, 2, 3, 4]. Despite advances in supportive care, effective pharmacological interventions remain elusive, largely due to the complexity of sepsis pathophysiology and the lack of therapies that address both immune and neural dysfunction [5, 6, 7]. Among the affected organs, the lung is particularly vulnerable, often serving as the first site of injury and a critical determinant of patient survival [8, 9]. Recent evidence indicates that sepsis‐associated inflammation compromises the peripheral nervous system, particularly within pulmonary tissues, where immune‐mediated neuronal damage contributes to respiratory symptoms such as chest pain, dyspnea, and impaired respiratory control [10]. Although peripheral neurons retain some regenerative capacity, this potential is markedly impaired under sustained inflammatory stress [11, 12]. In this context, neurotrophic factors‐critical regulators of neuronal survival and repair‐play an essential protective role but are insufficiently mobilized during sepsis.

Macrophages are central regulators of the septic inflammatory response [13]. These highly plastic cells can adopt a pro‐inflammatory M1 phenotype that amplifies tissue damage or switch to an anti‐inflammatory M2 phenotype that promotes repair and resolution [14, 15, 16]. In sepsis, sustained M1 polarization drives a cytokine storm that exacerbates tissue injury, while impaired M2 responses hinder recovery. Recent studies have revealed that macrophages engage in bidirectional crosstalk with local neurons, influencing neuronal survival through both cytokine release and neurotrophic factor secretion [17, 18, 19]. However, whether targeted modulation of macrophage phenotype can simultaneously resolve inflammation and protect neurons in septic lungs remains unclear.

Recent studies underscore the importance of immunometabolism in shaping macrophage phenotypes [20]. Notably, glutamate sustains M1 macrophage activation and drives pro‐inflammatory cytokine production [21, 22]. The glutamate production inhibitor JHU083 exhibits potent immunomodulatory activity by promoting M2‐like functions. Meanwhile, polyamines such as spermine (Spm) exert direct neuroprotective effects by stabilizing membranes, scavenging reactive oxygen species, and enhancing neurotrophic signaling [23, 24, 25]. These insights suggest that coordinated delivery of an immunometabolic modulator and a neuroprotective agent could achieve synergistic benefits in sepsis.

**FIGURE 1:**
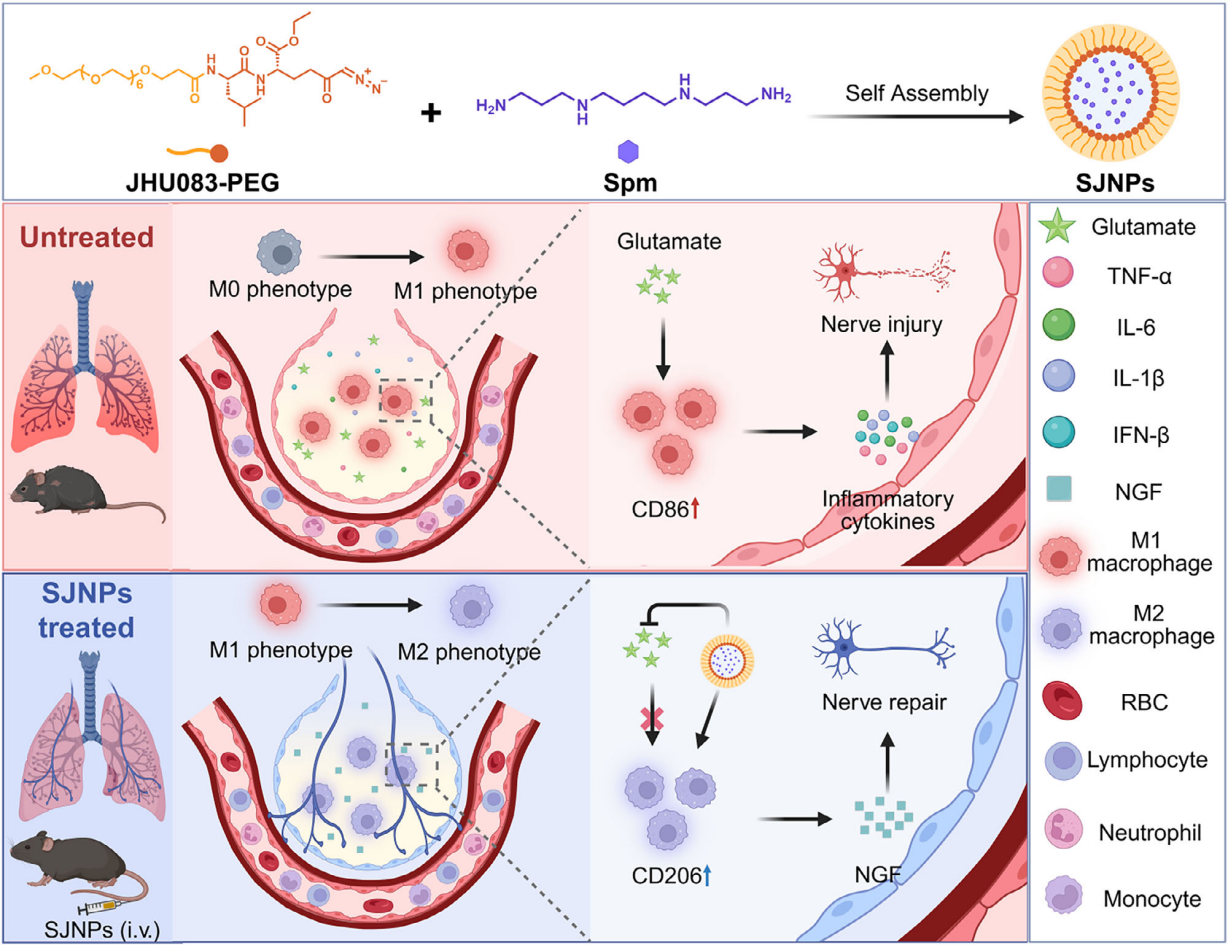
Schematic diagram illustrating the mechanism by which SJNPs alleviate sepsis‐induced lung injury and associated lung nerve damage. Created with BioRender.com.

To operationalize this concept, we report the development of an enzyme‐responsive, dual‐functional nanoplatform (SJNPs) designed to co‐deliver JHU083 and Spm for modulation of macrophage‐neuron immunometabolic crosstalk in sepsis. This design enables synchronized release within inflamed tissues, which reprograms macrophage polarization toward an anti‐inflammatory state, leading to enhanced nerve growth factor (NGF) secretion and protection of pulmonary neurons from inflammation‐induced injury. In murine sepsis models, SJNPs attenuated systemic and pulmonary inflammation, preserved lung neuronal integrity, and improved survival in a macrophage‐dependent manner. Our findings identify metabolic reprogramming of macrophage‐neuron crosstalk as a viable entry point for integrated immunomodulatory and neuroprotective therapy in sepsis, expanding the conceptual framework for treating organ‐specific injury in systemic inflammatory diseases (Figure 1).

## 2 Results

### 2.1 JHU083 Suppresses Pro‐Inflammatory Macrophage Polarization and Improves Survival in Murine Sepsis

**FIGURE 2:**
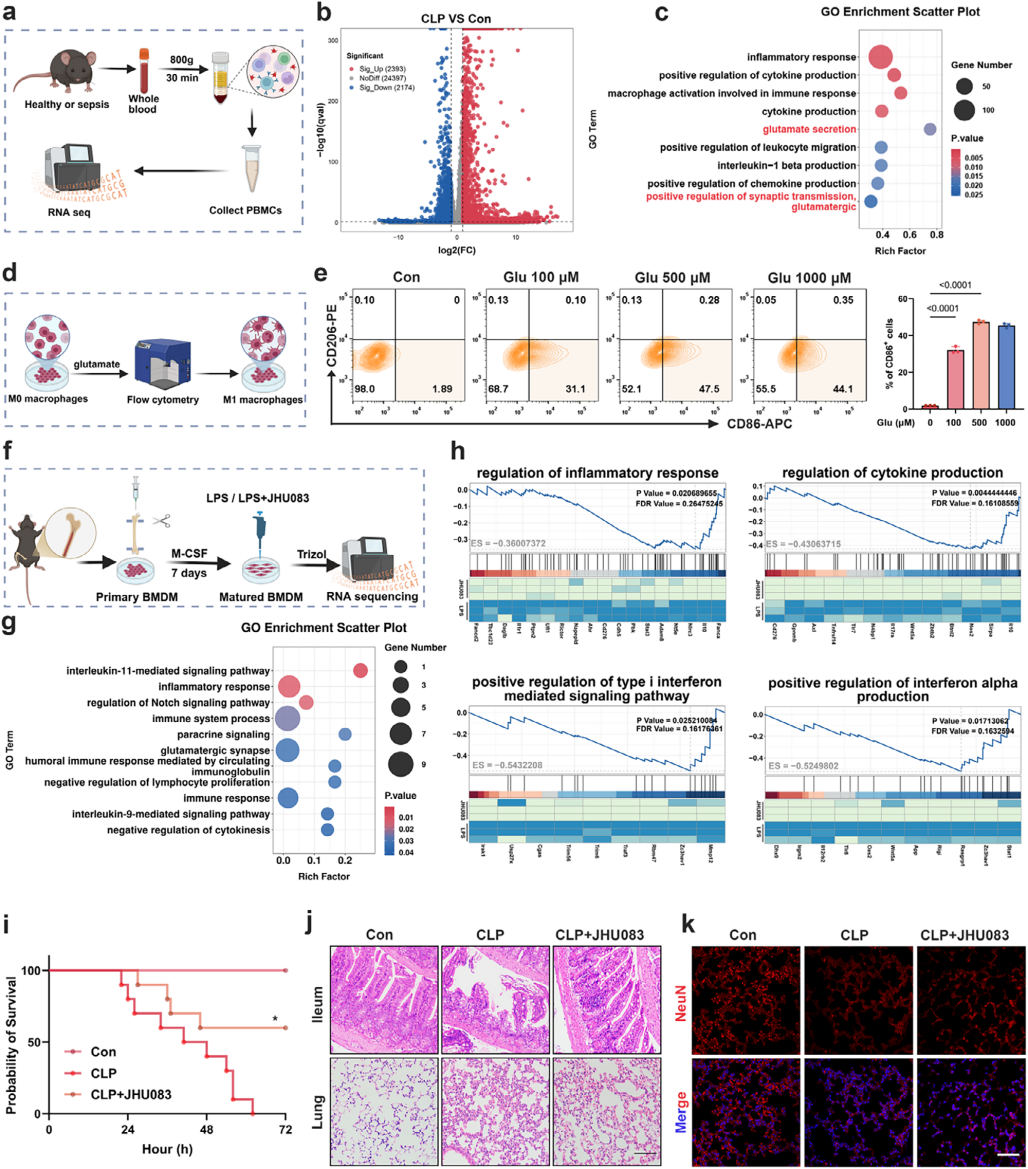
JHU083 suppresses pro‐inflammatory macrophage polarization and improves survival in murine sepsis. (a) Schematic of the RNA‐seq experimental design in CLP‐induced septic mice. (b) Volcano plot showing differentially expressed genes (DEGs) between the CLP group and control group (n = 3 per group). (c) GO enrichment analysis of DEGs identified in septic versus healthy mice. (d) Experimental scheme for glutamate stimulation in macrophages at various concentrations. (e) Flow cytometry analysis of macrophage polarization after 24 h glutamate treatment (n = 3 per group). (f) Primary BMDMs were derived from mouse bone marrow using M‐CSF (10 ng/mL, 7 days), stimulated with LPS (4 h), followed by JHU083 treatment for 24 h prior to RNA‐seq (n = 3 per group). (g) GO enrichment analysis of DEGs between JHU083‐treated and untreated BMDMs. (h) GSEA illustrating key pathways differentially regulated by JHU083 in BMDMs. (i) Kaplan‐Meier survival curves of CLP mice treated with PBS or JHU083 (n = 10 per group). (j) Representative H&E staining images of the ileum and lung tissues from each group. Scale bar, 100 µm. (k) Immunofluorescence staining of NeuN in lung sections. Scale bar, 50 µm. Error bars represent means ± SD. Differences between groups were tested using one‐way ANOVA followed by Tukey's multiple comparisons test, or unpaired Student's t‐test.

To gain mechanistic insight into the inflammatory cascade triggered by sepsis and to evaluate potential metabolic interventions, we employed a well‐established murine sepsis model using cecal ligation and puncture (CLP). Peripheral blood was collected 24 h post‐surgery, and peripheral blood mononuclear cells (PBMCs) were isolated for transcriptomic analysis by RNA sequencing (RNA‐seq) (Figure 2a). Compared with healthy controls, septic mice exhibited extensive transcriptional remodeling, with 2393 genes significantly upregulated and 2174 downregulated (Figure 2b). Gene Ontology (GO) enrichment analysis highlighted glutamate‐associated signaling pathways, such as glutamate secretion and positive regulation of synaptic transmission, glutamatergic, among the most significantly enriched biological processes (Figure 2c).

Given the established role of macrophages in orchestrating sepsis‐related inflammation, we next examined whether glutamate could directly influence macrophage polarization. In vitro stimulation of macrophages with glutamate drove a pronounced shift toward the pro‐inflammatory M1 phenotype (Figure 2d,e). To pharmacologically counteract this effect, bone marrow‐derived macrophages (BMDMs) were stimulated with lipopolysaccharide (LPS) and subsequently treated with JHU083, a glutamine antagonist that suppresses glutamate production, prior to RNA‐seq analysis (Figure 2f). GO analysis revealed that JHU083 markedly downregulated key inflammation‐related signaling pathways in LPS‐stimulated BMDMs (Figure 2g), and Gene Set Enrichment Analysis (GSEA) further confirmed broad suppression of pro‐inflammatory transcriptional programs (Figure 2h). These data indicate that JHU083 can effectively modulate macrophage polarization toward a less inflammatory phenotype.

We then assessed the in vivo therapeutic potential of JHU083 in the CLP model. JHU083 treatment significantly extended survival compared with vehicle‐treated septic mice (Figure 2i), indicating a systemic protective effect. Histopathological analysis showed that JHU083 substantially alleviated villus atrophy and mucosal injury in the ileum, but only modestly reduced inflammatory damage in the lungs (Figure 2j). Given the dense peripheral innervation and neuroimmune sensitivity of pulmonary tissue, we further examined neuronal integrity by immunofluorescence staining for neural markers. These analyses revealed that JHU083 provided limited protection against sepsis‐induced pulmonary neuronal injury (Figure 2k).

In summary, JHU083 suppresses inflammatory signaling pathways and prolongs survival in murine sepsis. However, its limited efficacy in mitigating lung‐specific inflammation and associated neuronal damage highlighted the need for a combinatorial approach integrating both immunomodulation and neuroprotection.

### 2.2 Design and Physicochemical Characterization of SJNPs for Synergistic Delivery of JHU083 and Spm

**FIGURE 3:**
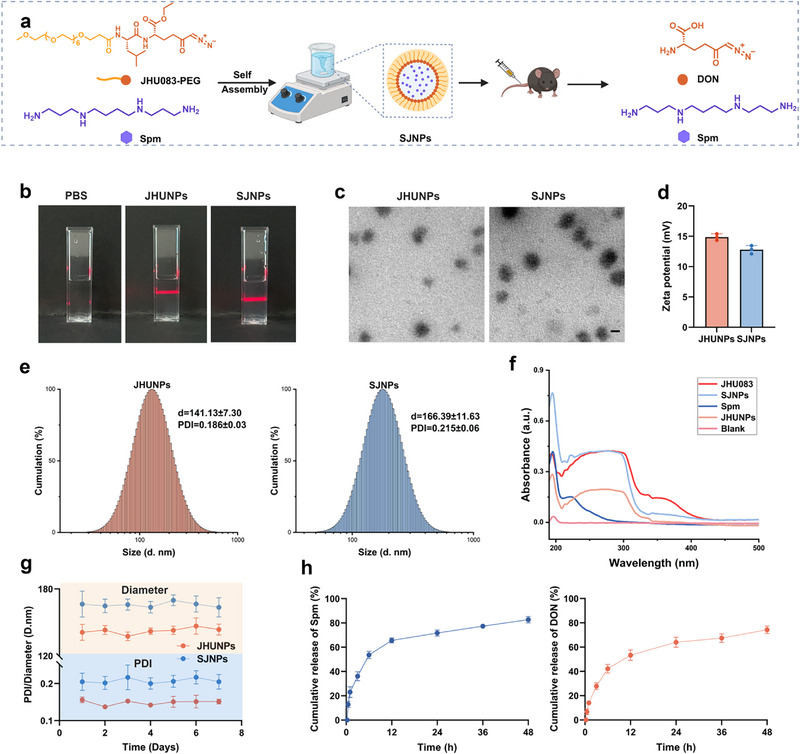
Design and physicochemical characterization of SJNPs for synergistic delivery of JHU083 and spermine. (a) Schematic illustration of the self‐assembly process and drug release mechanism of SJNPs. (b) Visualization of the Tyndall effect indicating colloidal stability of the nanoparticle suspension. (c) Representative TEM images of JHUNPs and SJNPs. Scale bars, 100 nm. (d) Particle size distribution of JHUNPs and SJNPs measured by DLS. (e) Zeta potential analysis of JHUNPs and SJNPs. (f) UV–vis absorption spectra of JHUNPs and SJNPs. (g) The changes in particle size and PDI of JHUNPs and SJNPs in PBS over time reflect their colloidal stability. (h) The cumulative drug release profile of SJNPs after incubation with serum was measured by HPLC. Error bars represent means ± SD. Differences between groups were tested using one‐way ANOVA followed by Tukey's multiple comparisons test, or unpaired Student's t‐test.

Given the limited efficacy of JHU083 in protecting against sepsis‐induced pulmonary and neuronal damage, we sought to enhance its therapeutic performance by incorporating Spm, a polyamine with well‐documented anti‐inflammatory and neuroprotective properties. We first modified JHU083 with polyethylene glycol (PEG) to facilitate its self‐assembly into JHU083 nanoparticles (JHUNPs). The successful synthesis of the JHU083‐PEG conjugate was validated by ^1^H NMR, ^13^C NMR, and mass spectrometry (MS) (Scheme S1). To enable synchronized delivery and targeted release of both agents, we designed a self‐assembled nanocarrier system Spm@JHU083‐PEG nanoparticles (SJNPs). This platform was engineered to respond to the enzymatic milieu of inflamed tissues, thereby achieving controlled and synergistic drug release. Specifically, amidase‐, esterase‐mediated, and proteinase K cleavage of the PEG shell triggers the liberation of active DON (the pharmacophore of JHU083), while concurrent disassembly of the nanoparticle matrix releases encapsulated Spm (Figure 3a).

To confirm the self‐assembly behavior of SJNPs, Tyndall effect assays revealed pronounced light scattering indicative of stable colloidal dispersion for both JHUNPs and SJNPs (Figure 3b). Transmission electron microscopy (TEM) further confirmed the formation of uniform, spherical nanostructures (Figure 3c). Physicochemical characterization demonstrated favorable surface charges, with zeta potentials of +14.88 mV for JHUNPs and +12.80 mV for SJNPs (Figure 3d), indicative of good colloidal stability. Dynamic light scattering (DLS) analysis revealed average hydrodynamic diameters of 141.13 nm for JHUNPs and 166.39 nm for SJNPs, with low polydispersity indices (PDI) of 0.186 and 0.215, respectively (Figure 3e). UV–visible (UV–vis) spectroscopy confirmed the successful co‐assembly of JHU083 and Spm within a single nanoplatform, without evidence of phase separation or aggregation (Figure 3f).

To evaluate long‐term colloidal stability, particle size and PDI were monitored in PBS over 7 days, showing no significant changes in either parameter (Figure 3g). Furthermore, high‐performance liquid chromatography (HPLC) analysis of serum samples from CLP mice demonstrated sustained and efficient drug release, with cumulative release rates of 82.74% for Spm and 74.13% for DON within 48 h post‐administration (Figure 3h). Together, these data establish SJNPs as a stable, enzyme‐responsive nanoplatform capable of co‐delivering JHU083 and Spm in a controlled manner, providing a robust foundation for subsequent in vivo therapeutic evaluation in sepsis models.

### 2.3 SJNPs Mitigate Systemic Inflammation and Prolong Survival in Sepsis Models

**FIGURE 4:**
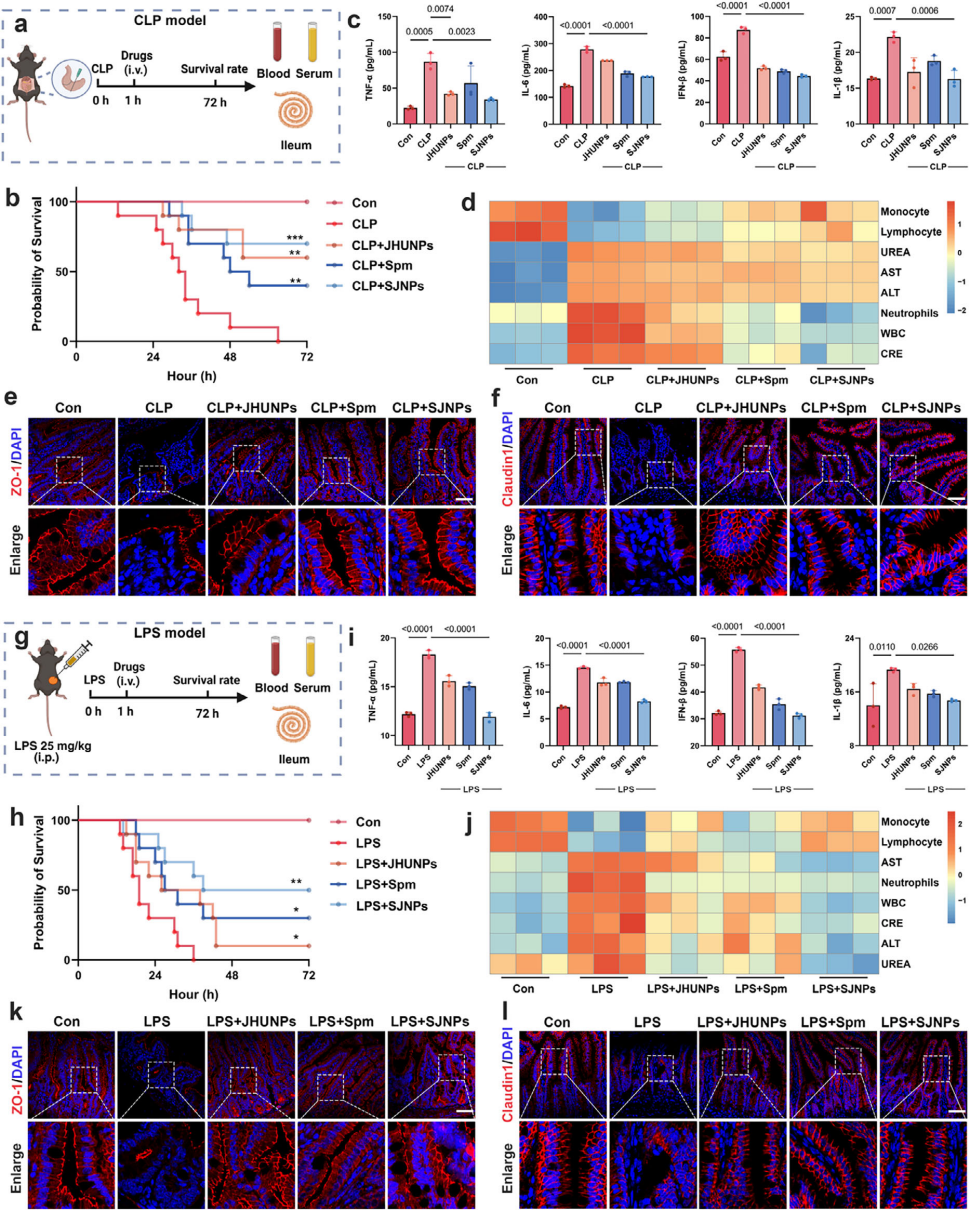
SJNPs mitigate systemic inflammation and prolong survival in sepsis models. (a) Schematic representation of CLP model construction. (b) Survival curves of septic mice in the CLP model following treatment with PBS, JHUNPs (0.5 mg/kg), Spm (1 mg/kg), or SJNPs (1.3 mg/kg) (n = 10 per group). (c) Quantification of pro‐inflammatory cytokines (TNF‐α, IL‐6, IL‐1β, and IFN‐β) in serum samples by ELISA in the CLP model (n = 3 per group). (d) Heatmaps of hematological and biochemical parameters in the CLP model. Hematological indices include white blood cells (WBC), neutrophils, monocytes, and lymphocytes. Biochemical markers include alanine aminotransferase (ALT), aspartate aminotransferase (AST), creatinine (CRE), and blood urea nitrogen (UREA). Values were normalized to the mean of the control group (n = 3 per group). (e, f) Representative immunofluorescence images of ileum sections stained for tight junction proteins ZO‐1 and Claudin1 in the CLP model. Scale bars, 100 µm. (g) Schematic representation of LPS model construction. (h) Survival curves of septic mice in the LPS model following treatment with PBS or SJNPs (n = 10 per group). (i) Quantification of TNF‐α, IL‐6, IL‐1β, and IFN‐β in serum by ELISA in the LPS model (n = 3 per group). (j) Heatmaps of hematological and biochemical indicators in the LPS model (n = 3 per group). (k, l) Representative immunofluorescence images of ileum sections stained with ZO‐1 and Claudin1 in the LPS model. Scale bars, 100 µm. Error bars represent means ± SD. Differences between groups were tested using one‐way ANOVA followed by Tukey's multiple comparisons test, or unpaired Student's t‐test.

To evaluate the in vivo therapeutic potential of SJNPs, we employed two complementary murine sepsis models: CLP model and LPS model [26, 27]. In the classical polymicrobial CLP model, SJNPs treatment markedly extended survival compared with either monotherapy (Figure 4a,b), prompting a detailed assessment of their systemic anti‐inflammatory and organ‐protective effects. Serum cytokine profiling by Enzyme‐linked immunosorbent assay (ELISA) revealed that SJNPs significantly lowered pro‐inflammatory mediators, which include tumor necrosis factor‐alpha (TNF‐α), interleukin‐6 (IL‐6), interferon‐beta (IFN‐β), and interleukin‐1 beta (IL‐1β), indicating a robust suppression of systemic inflammation (Figure 4c). Comprehensive hematological and biochemical analyses further substantiated these effects: complete blood count (CBC) data showed reduced circulating white blood cells and neutrophils, alongside increased monocyte and lymphocyte populations (Figure S1a). In parallel, serum biochemistry demonstrated reduced hepatic (AST, ALT, ALP) and renal (UREA, UA, CRE) injury markers, supporting both hepatoprotective and nephroprotective roles (Figure S1b). These systemic improvements were visualized via heatmaps, providing an integrated view of SJNPs‐induced physiological recovery (Figure 4d). Given that intestinal barrier disruption is a major driver of systemic inflammation in sepsis, we next examined SJNPs’ effects on gut integrity. Hematoxylin‐eosin staining (H&E) revealed that SJNPs ameliorated villus atrophy and structural damage in the ileum of CLP mice (Figure S1c). Immunofluorescence and Western blot analyses confirmed restoration of tight junction proteins ZO‐1 and Claudin1, which were otherwise downregulated in septic conditions, indicating preserved epithelial barrier function (Figure 4e,f; Figure S1D).

To confirm reproducibility and model‐independence, we validated these findings in an LPS‐induced endotoxemia model (Figure 4g). Consistent with the CLP results, SJNPs significantly prolonged survival and outperformed either monotherapy (Figure 4h). ELISA assays again confirmed systemic suppression of TNF‐α, IL‐6, IFN‐β, and IL‐1β (Figure 4i), and CBC profiles mirrored the CLP trends, with reduced counts of white blood cells and neutrophils, and elevated counts of monocytes and lymphocytes (Figure S1e). Similarly, liver and kidney injury markers were significantly decreased (Figure S1f), and heatmaps illustrated broad systemic recovery (Figure 4j). Histological and molecular assessments of the ileum reinforced these protective effects: H&E staining demonstrated reduced villus injury (Figure S1g), and ZO‐1 and Claudin1 expression was restored (Figure 4k,l; Figure S1h), indicating maintenance of gut barrier integrity. Importantly, toxicity evaluation confirmed no discernible histopathological damage in major organs, supporting the biosafety of SJNPs for systemic use (Figure S2).

In summary, SJNPs not only substantially prolong survival in sepsis models but also attenuate systemic inflammation, protect organ function, and preserve intestinal barrier integrity, underscoring their promise as a therapeutic strategy against sepsis‐induced systemic injury.

### 2.4 SJNPs Attenuate Sepsis‐Induced Pulmonary Injury and Restore Organ Homeostasis

**FIGURE 5:**
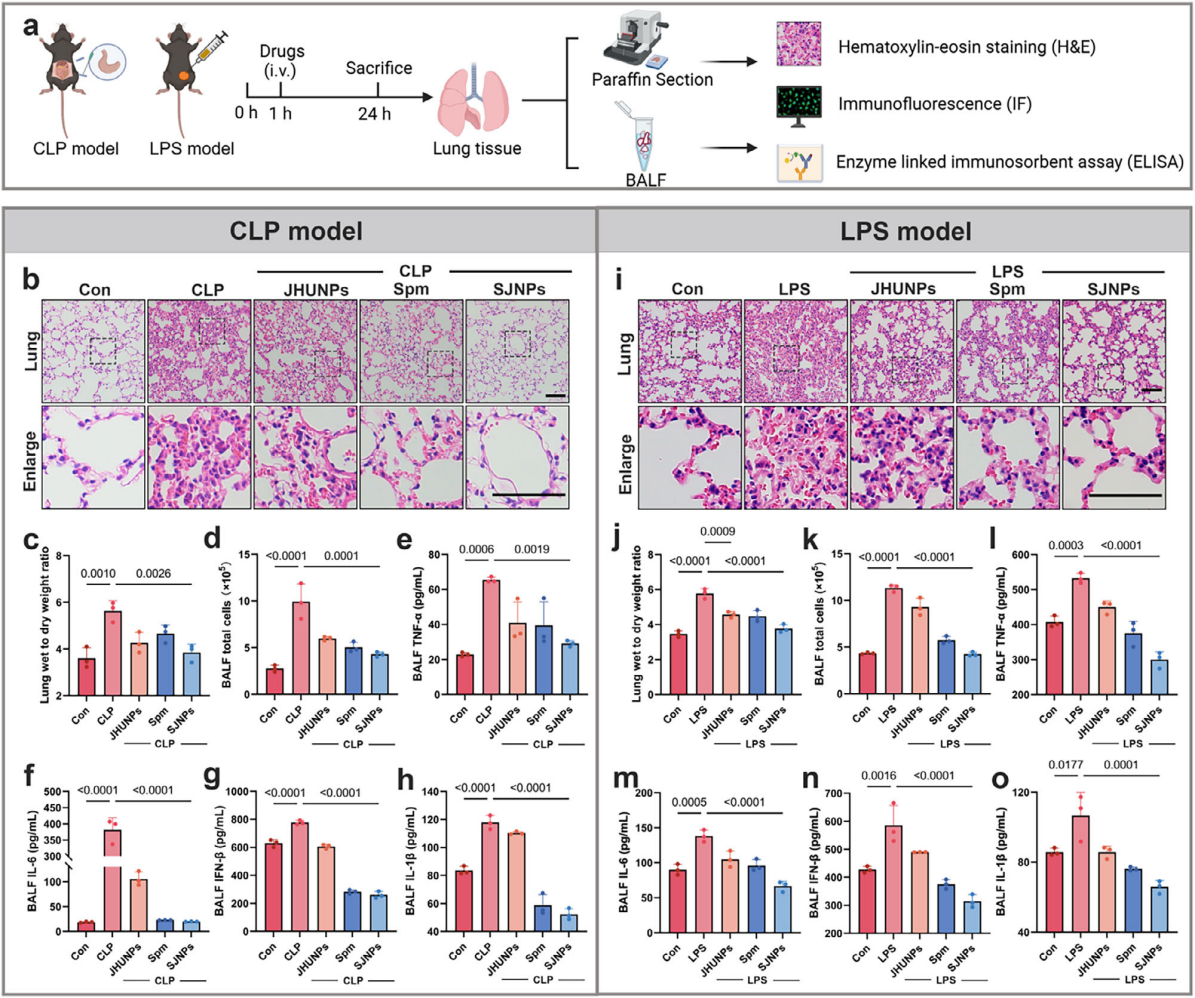
SJNPs attenuate sepsis‐induced pulmonary injury and restore organ homeostasis. (a) Schematic representation of the treatment protocol for lung tissue analysis in the sepsis model. (b) Representative H&E staining images of lung tissue from CLP‐induced septic mice. Scale bar, 100 µm. (c) Lung wet‐to‐dry weight ratio in the CLP model (n = 3 per group). (d) Total cell count in BALF from the CLP model (n = 3 per group). (e–h) Levels of TNF‐α, IL‐6, IL‐1β, and IFN‐β in BALF measured by ELISA in the CLP model (n = 3 per group). (i) Representative H&E staining of lung tissue from LPS‐induced septic mice. Scale bar, 100 µm. (j) Lung wet‐to‐dry weight ratio in the LPS model (n = 3 per group). (k) Total cell count in BALF from the LPS model (n = 3 per group). (l–o) ELISA quantification of TNF‐α, IL‐6, IL‐1β, and IFN‐β in BALF from LPS model mice (n = 3 per group). Error bars represent means ± SD. Differences between groups were tested using one‐way ANOVA followed by Tukey's multiple comparisons test, or unpaired Student's t‐test.

As one of the most vulnerable organs during sepsis, the lung often represents the initial site of organ failure and a major determinant of mortality [28, 29, 30]. in vivo fluorescence imaging revealed enhanced accumulation of JHUNPs and SJNPs in the lungs of septic mice (Figure S3a). Consistently, *ex vivo* imaging of isolated major organs (heart, liver, spleen, lung, and kidney) further validated the preferential localization of JHUNPs and SJNPs in lung tissue (Figure S3b,c). To evaluate the protective role of SJNPs against sepsis‐induced pulmonary injury, we performed integrated histological, molecular, and cellular analyses in both CLP and LPS models (Figure 5a). In the CLP model, H&E staining showed that lungs from SJNPs‐treated mice preserved more intact alveolar architecture and exhibited markedly reduced inflammatory cell infiltration compared with untreated controls (Figure 5b), indicating substantial mitigation of tissue injury. Consistently, SJNPs administration significantly decreased the lung wet to dry weight ratio (Figure 5c), reflecting a potent anti‐edematous effect.

To further probe local inflammatory responses, we analyzed bronchoalveolar lavage fluid (BALF) [31, 32]. Total cell counts in BALF, which were markedly elevated in septic mice, were significantly reduced following SJNPs treatment (Figure 5d). ELISA analysis revealed that SJNPs markedly suppressed BALF concentrations of TNF‐α, IL‐6, IFN‐β, and IL‐1β (Figure 5e–h), aligning with the systemic anti‐inflammatory effects observed in serum. These findings confirm that SJNPs exert strong local anti‐inflammatory activity within the septic lung.

To confirm these effects in an independent model, we tested SJNPs in LPS‐induced sepsis. Histological examination again revealed preserved alveolar structure and reduced inflammatory infiltration in SJNPs‐treated lungs (Figure 5i), accompanied by significantly decreased lung wet to dry ratios (Figure 5j). BALF analysis confirmed reductions in total cell count (Figure 5k) and pro‐inflammatory cytokines (TNF‐α, IL‐6, IFN‐β, IL‐1β) (Figure 5l–o). Taken together, these results highlight the ability of SJNPs to attenuate sepsis‐induced lung injury through multi‐faced mechanisms‐namely, suppression of cytokine‐driven inflammation, reduction of pulmonary edema, and macrophage phenotype reprogramming toward a reparative state.

### 2.5 SJNPs Reprogram Macrophage Polarization Toward an Anti‐Inflammatory Phenotype

Building on their protective effects against lung injury, we next investigated whether the therapeutic benefits of SJNPs are mediated through the regulation of macrophage polarization. Macrophages are central orchestrators of the inflammatory microenvironment and exist in functionally distinct phenotypic states. Thus, polarization dynamics represent a plausible mechanistic axis for their action [33, 34].

**FIGURE 6:**
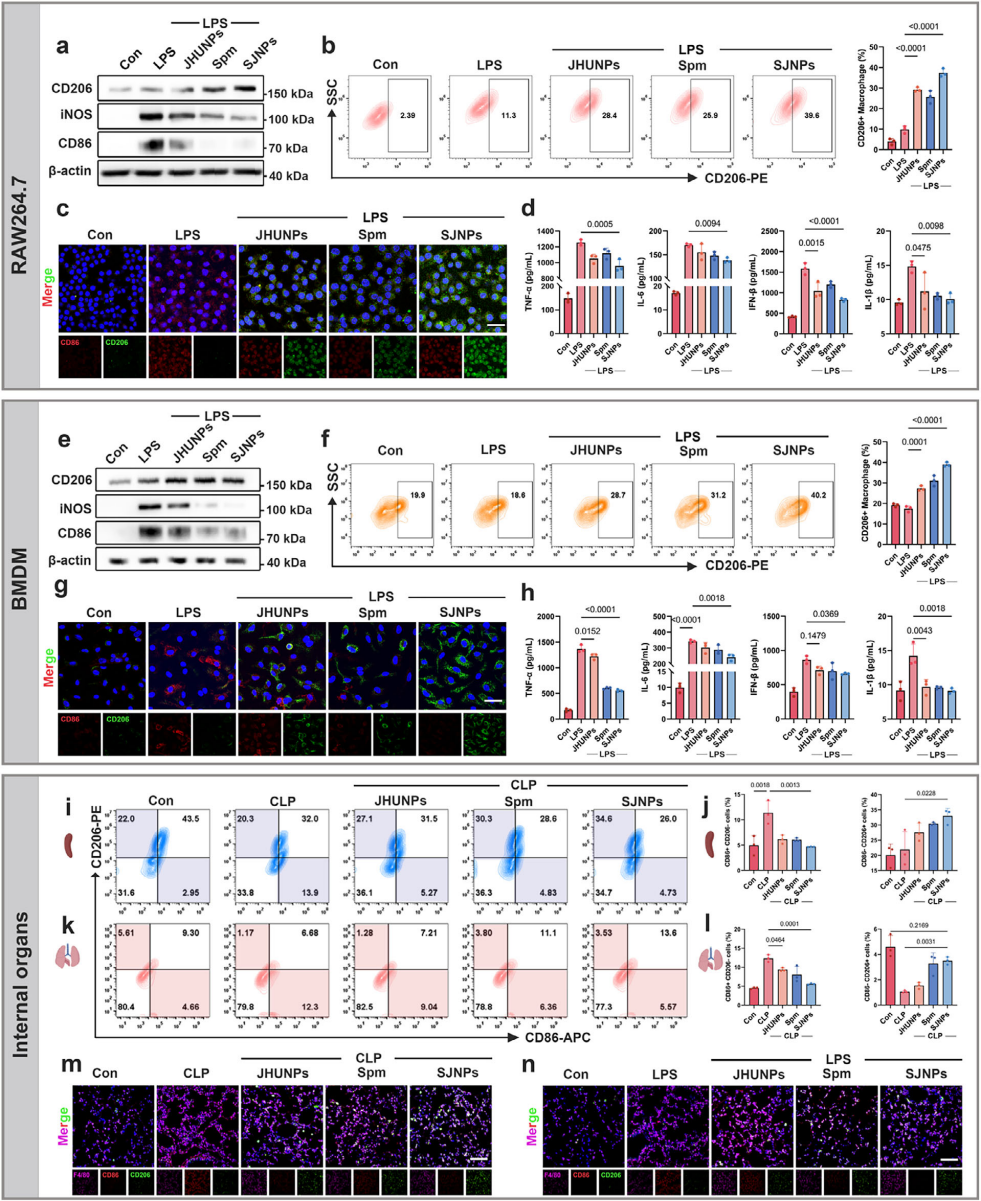
SJNPs reprogram macrophage phenotype to resolve systemic inflammation. (a) Western blot analysis of polarization‐associated marker proteins (CD86 and CD206) in RAW264.7 cells following LPS and drug treatments (JHUNPs, 5 µM; Spm, 35 µM), with β‐actin used as a loading control. (b) Flow cytometric analysis of CD206 expression in RAW264.7 cells. (c) Immunofluorescence staining of CD86 and CD206 in RAW264.7 cells under different polarization conditions. Scale bar, 30 µm. (d) ELISA quantification of TNF‐α, IL‐6, IL‐1β, and IFN‐β levels in the supernatant of RAW264.7 cells (n = 3 per group). (e) Western blot analysis of CD86 and CD206 expression in BMDM cells treated with LPS and drugs, with β‐actin as a loading control. (f) Flow cytometric analysis of CD206 expression in BMDM cells following polarization. (g) Immunofluorescence staining of CD86 and CD206 in BMDM cells under different polarization conditions. Scale bar, 30 µm. (h) ELISA quantification of TNF‐α, IL‐6, IL‐1β, and IFN‐β levels in the supernatant of BMDM cells (n = 3 per group). (i–l) Flow cytometric analysis of CD86 and CD206 expression in major macrophage‐resident organs, including the spleen and lung, from septic mice with or without SJNPs treatment. (m) Immunofluorescence staining of macrophages in lung tissue from CLP mice. Scale bar, 50 µm. (n) Immunofluorescence staining of macrophages in lung tissue from LPS model mice. Scale bar, 50 µm. Error bars represent means ± SD. Differences between groups were tested using one‐way ANOVA followed by Tukey's multiple comparisons test, or unpaired Student's t‐test.

We first examined macrophage phenotype shifts in vitro using the murine macrophage cell line RAW264.7. Upon LPS stimulation, RAW264.7 cells exhibited elevated expression of the M1 phenotype surface marker CD86, indicative of a pro‐inflammatory state [35]. SJNPs treatment markedly reduced CD86 expression while concomitantly increasing CD206, a canonical marker of the anti‐inflammatory M2 phenotype, as confirmed by Western blot, flow cytometry, and immunofluorescence (Figure 6a–c). Since macrophage‐driven inflammation is largely propagated through cytokine release, we quantified supernatant cytokines by ELISA and found that SJNPs significantly suppressed TNF‐α, IL‐6, IFN‐β, and IL‐1β secretion (Figure 6d). To validate these results in a more physiologically relevant context, we repeated the experiments using BMDMs. Consistent with RAW264.7 findings, LPS stimulation induced robust CD86 expression in BMDMs, which was effectively suppressed by SJNPs, while CD206 expression was enhanced (Figure 6e–g). ELISA analysis again confirmed substantial reductions in TNF‐α, IL‐6, IFN‐β, and IL‐1β secretion (Figure 6h), underscoring the immunomodulatory activity of SJNPs.

We next examined whether this polarization shift occurs in vivo by performing flow cytometry on macrophage‐enriched organs from CLP mice, including spleen, lung, and ileum [36]. Across all tissues, sepsis induced a marked increase in CD86^+^ macrophages, which was reversed by SJNPs, accompanied by a parallel rise in CD206 expression (Figure 6i–l; Figure S4a,b), indicating systemic macrophage reprogramming. Given the pivotal role of macrophage polarization in modulating pulmonary inflammation, we next examined macrophage phenotypes in lung tissue in both the CLP and LPS models. Immunofluorescence staining demonstrated that SJNPs inhibited M1 pro‐inflammatory polarization while promoting the reparative M2 phenotype, indicating immune microenvironment reprogramming toward tissue repair (Figure 6m,n).

Collectively, these in vitro and in vivo results demonstrate that SJNPs exert a robust and consistent effect on macrophage polarization, driving a shift toward the anti‐inflammatory M2 state. This phenotypic switch contributes to cytokine suppression and inflammation resolution, positioning SJNPs as a promising immunotherapeutic strategy for sepsis‐associated inflammation.

### 2.6 SJNPs Preserve Pulmonary Neuronal Integrity in Septic Conditions

Beyond immune modulation, sepsis also compromises pulmonary neuroarchitecture, impairing respiratory regulation [37, 38, 39]. Neurons within lung tissue coordinate bronchomotor tone, vascular regulation, and gas‐exchange reflexes. Excessive inflammatory mediators, such as TNF‐α, IL‐6, IFN‐β, and IL‐1β can trigger neuronal apoptosis and fibrosis, leading to functional decline. Thus, preserving lung neuronal integrity is essential for maintaining respiratory homeostasis during sepsis [40].

**FIGURE 7:**
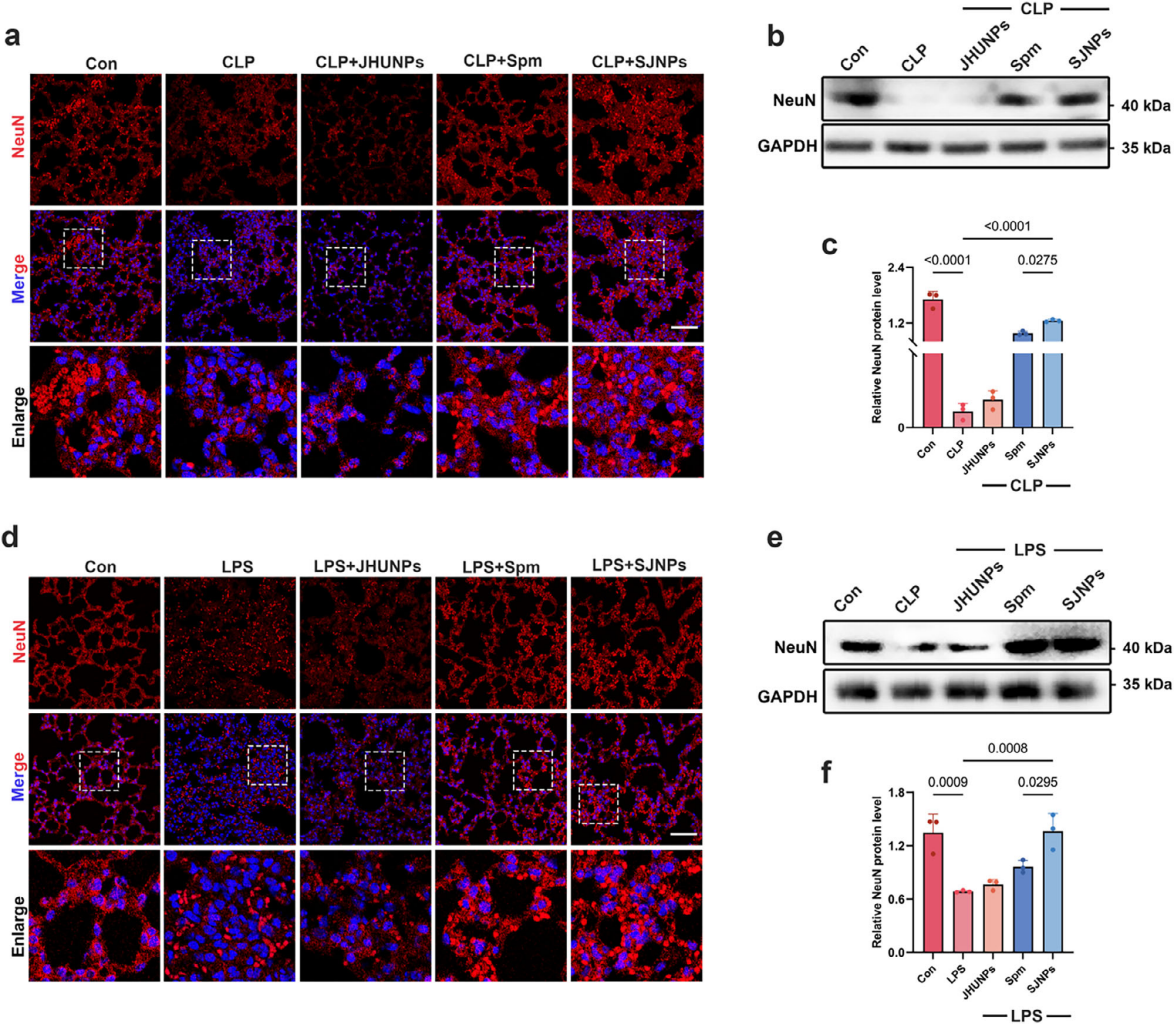
SJNPs protect pulmonary neurons from inflammation‐induced damage in sepsis. (a–c) Immunofluorescence staining and Western blot analysis of NeuN expression in lung tissues from the CLP model. Scale bar, 50 µm. (d–f) Immunofluorescence staining and Western blot analysis of NeuN expression in lung tissues from the LPS model. Scale bar, 50 µm. Error bars represent means ± SD. Differences between groups were tested using one‐way ANOVA followed by Tukey's multiple comparisons test, or unpaired Student's t‐test.

To determine whether SJNPs confer neuroprotection in septic lungs, we assessed NeuN, a well‐established marker of mature neurons, by immunofluorescence in CLP mice. Compared to vehicle‐treated animals, SJNPs‐treated mice displayed significantly greater NeuN‐positive signal, indicating reduced neuronal loss (Figure 7a). Western blot analysis corroborated these results, showing higher NeuN protein levels in SJNPs‐treated lungs relative to untreated CLP controls (Figure 7b,c). We confirmed these findings in the LPS‐induced sepsis model. Both immunofluorescence and Western blot analyses demonstrated preservation of NeuN expression following SJNPs treatment (Figure 7d–f), indicating that the neuroprotective effects are not model‐restricted.

Together, these data demonstrate that SJNPs alleviate sepsis associated neuronal injury in the lungs, likely by dampening inflammation‐induced neurotoxicity, thereby preserving critical neural elements.

### 2.7 Macrophage‐Neuron Crosstalk is Essential for SJNPs‐Mediated Neuroprotection

**FIGURE 8:**
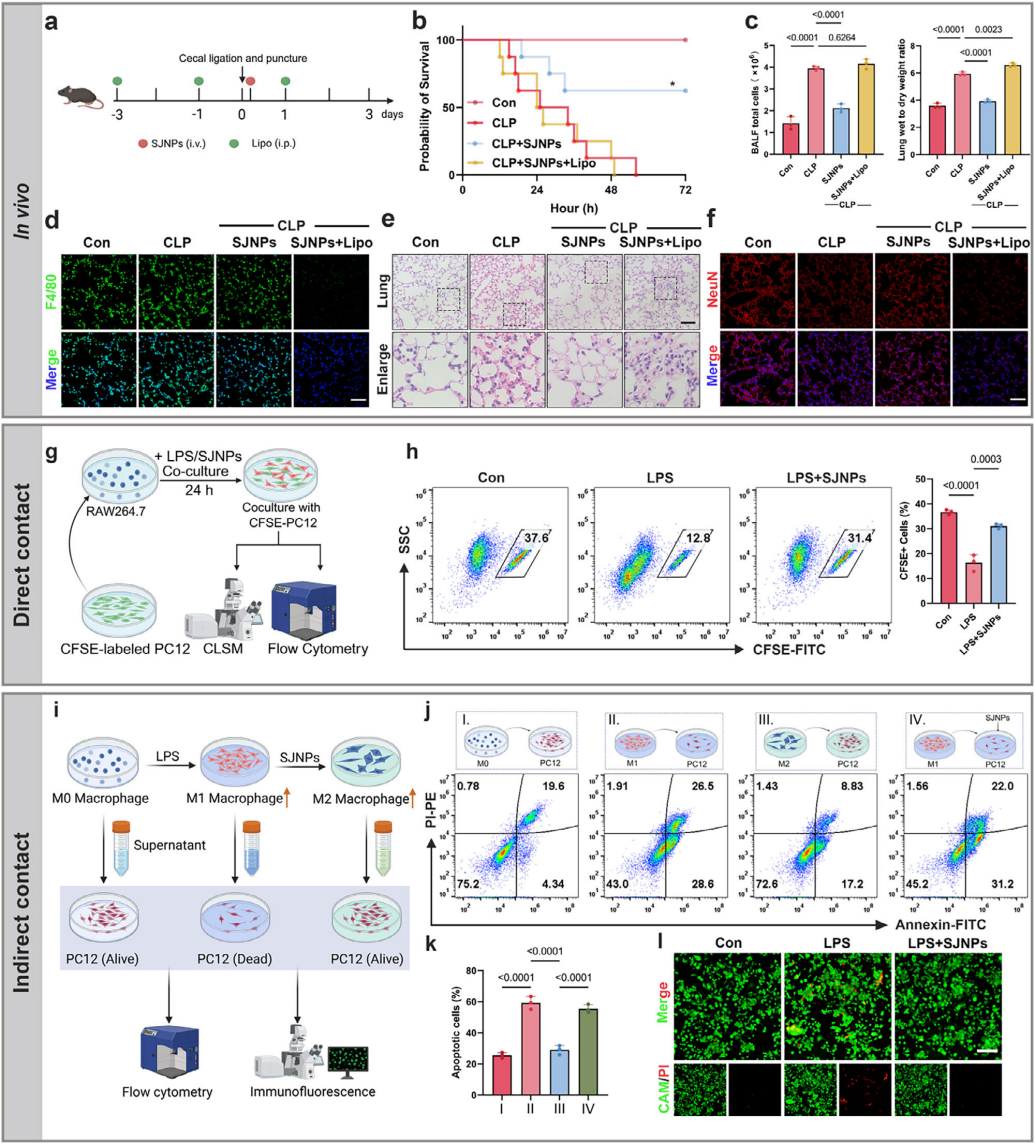
Macrophage‐neuron crosstalk drives SJNPs‐induced neuroprotection during sepsis. (a) Schematic of in vivo macrophage depletion assay. C57BL/6 mice were intraperitoneally injected with clodronate liposomes (Lipo, 200 µL/mouse). (b) Survival was monitored for 72 h after CLP induction and treatment with or without SJNPs in macrophage‐depleted or control mice (n = 8 per group). (c) Lung wet‐to‐dry ratio (left) and total cell count in BALF (right) from the CLP model (n = 3 per group). (d) Immunofluorescence staining of the macrophage marker F4/80 in lung tissue. Scale bar, 50 µm. (e) Representative H&E‐stained sections of lung tissue from the CLP model. Scale bar, 100 µm. (f) Immunofluorescence staining of the neuronal marker NeuN in lung tissue. Scale bar, 50 µm. (g) Schematic of the direct co‐culture experiment: CFSE‐labeled PC12 cells were co‐cultured with RAW264.7 macrophages and simultaneously exposed to LPS or SJNPs for 24 h. Neuronal viability was subsequently assessed using flow cytometry and confocal fluorescence microscopy. (h) Quantification of viable CFSE‐labeled PC12 cells by flow cytometry after direct co‐culture with LPS or SJNPs‐treated RAW264.7 macrophages for 24 h. (i) Schematic of the indirect conditioned medium experiment: RAW264.7 macrophages were treated with LPS or SJNPs for 24 h, after which the culture medium was replaced with serum‐free medium for an additional 24 h. The collected supernatants were transferred to PC12 cells for 24 h to assess neuronal apoptosis via flow cytometry and immunofluorescence staining. (j,k) Apoptosis of PC12 cells assessed by flow cytometry after exposure to supernatants from differently treated macrophages. (l) Live/dead cell staining of PC12 cells after treatment with various RAW264.7‐derived supernatants. Scale bar, 200 µm. Error bars represent means ± SD. Differences between groups were tested using one‐way ANOVA followed by Tukey's multiple comparisons test, or unpaired Student's t‐test.

Emerging evidence highlights a critical crosstalk between the immune and nervous systems, wherein macrophages modulate the local inflammatory milieu via cytokine secretion, thereby influencing neuronal integrity [41]. In the lungs, tissue‐resident macrophages are essential for maintaining homeostasis under both physiological and pathological conditions. Given this context, we hypothesized that the neuroprotective effects of SJNPs in sepsis are critically dependent on macrophage presence and function. To test this, we selectively depleted macrophages in vivo using clodronate liposomes (Lipo), administered intraperitoneally prior to SJNPs treatment in the CLP model (Figure 8a). Consistent with previous findings, SJNPs markedly improved the survival of septic mice. However, this benefit was completely abolished following macrophage depletion (Figure 8b), indicating that macrophages are indispensable for SJNPs efficacy. We next assessed lung inflammation and injury markers. SJNPs significantly reduced BALF total cell counts and lung wet to dry ratios, which are two indices of pulmonary inflammation and edema, but these effects were entirely lost in macrophage‐depleted mice (Figure 8c). Immunofluorescence and H&E confirmed both efficient macrophage depletion and the loss of SJNPs‐mediated anti‐inflammatory protection in lung tissue (Figure 8d,e). Interestingly, histological evaluation of the ileum revealed that the systemic anti‐inflammatory benefits of SJNPs on the gut were also macrophage‐dependent (Figure S5a). Moreover, NeuN immunofluorescence demonstrated that the preservation of lung neuronal integrity by SJNPs was abrogated in the absence of macrophages (Figure 8f), directly linking SJNPs‐mediated neuroprotection to macrophage activity.

To dissect this link mechanistically, we employed direct and indirect co‐culture systems using PC12 neuronal cells and RAW264.7 macrophages. In direct‐contact cultures, CFSE‐labeled PC12 cells were co‐cultured with macrophages and treated with LPS and SJNPs (Figure 8g). Flow cytometry and immunofluorescence analysis showed that LPS significantly reduced PC12 viability, while SJNPs treatment restored it (Figure 8h; Figure S5b). To distinguish effects dependent on cell contact from those mediated by soluble factors, we performed indirect co‐culture using macrophage‐conditioned media (Figure 8i). Supernatants from SJNPs‐treated macrophages markedly reduced PC12 apoptosis, whereas direct exposure of PC12 cells to SJNPs in M1‐conditioned media failed to replicate this effect (Figure 8j,k), excluding a direct neuronal action of SJNPs. Live/dead staining further confirmed the neuroprotective effect of SJNPs conditioned macrophage supernatants (Figure 8l).

Together, these in vivo and in vitro results identify macrophages as essential intermediaries in SJNPs‐induced neuroprotection. By reprogramming macrophage inflammatory responses, SJNPs indirectly preserve neuronal viability in the lung, revealing a novel immune‐neural axis of therapeutic relevance.

### 2.8 NGF Induction by Reprogrammed Macrophages Mediates SJNPs‐Driven Neuronal Survival

**FIGURE 9:**
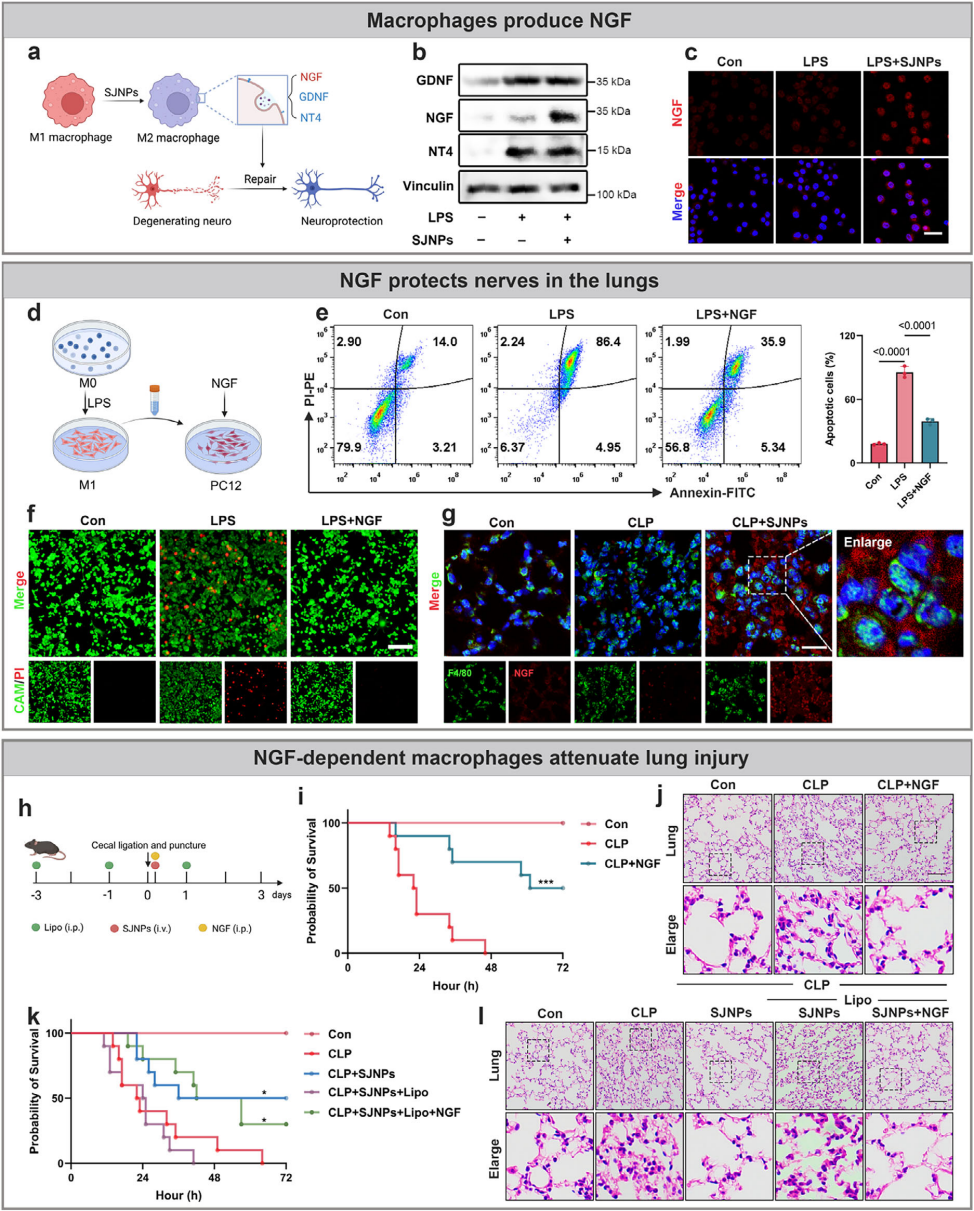
NGF secretion by reprogrammed macrophages underlies SJNPs‐induced neuroprotection. (a) Schematic illustration showing that macrophages promote neuronal repair through the release of neurotrophic factors. (b) Western blot analysis of neurotrophic factor expression in macrophages treated with LPS and SJNPs, with Brefeldin A (BFA) added 5 h prior to cell harvest. (c) Immunofluorescence staining of NGF expression in macrophages after SJNPs exposure. Scale bar, 30 µm. (d) Supernatants from LPS‐treated M0 macrophages were supplemented with exogenous NGF and added to PC12 cells for 24 h. (e) Flow cytometric analysis of PC12 apoptosis following treatment with NGF and macrophage supernatants. (f) Live/dead staining assay to assess apoptosis in PC12 cells after NGF treatment. Scale bar, 200 µm. (g) Immunofluorescence detection of NGF expression in lung‐resident macrophages from the CLP model. Scale bar, 20 µm. (h) in vivo macrophage depletion strategy: C57BL/6 mice were intraperitoneally injected with clodronate liposomes (200 µL per mouse) and subsequently treated with SJNPs and/or NGF. (i) Survival analysis of CLP model mice treated with NGF (n = 10 per group). (j) Representative H&E staining images of lung tissue from CLP mice treated with NGF. Scale bar, 100 µm. (k) Survival analysis of macrophage‐depleted CLP model mice receiving SJNPs and/or NGF (n = 10 per group). (l) Representative H&E staining of lung tissues showing the effect of NGF in the presence or absence of macrophages. Error bars represent means ± SD. Differences between groups were tested using one‐way ANOVA followed by Tukey's multiple comparisons test, or unpaired Student's t‐test.

Neurotrophic factors, which include NGF, glial cell‐derived neurotrophic factor (GDNF), and neurotrophin‐4 (NT‐4), are key regulators of neuronal survival, and their expression is dynamically modulated during inflammation. [42] To determine whether SJNPs‐induced neuroprotection involves macrophage‐derived neurotrophic signaling, we profiled these factors in macrophages. Western blot analysis revealed that SJNPs selectively upregulated NGF, but not GDNF or NT‐4 (Figure 9a,b). Immunofluorescence further confirmed elevated NGF expression in SJNPs‐treated macrophages (Figure 9c), indicating that SJNPs enhance neurotrophic signaling via NGF induction.

To assess sufficiency, we supplemented PC12 cells with recombinant NGF in the presence of conditioned media from M1‐polarized macrophages. NGF markedly reduced PC12 apoptosis in this inflammatory context (Figure 9d,e), as confirmed by live/dead staining (Figure 9f). in vivo, immunofluorescence of lung tissue from CLP mice confirmed increased NGF expression in lung‐resident macrophages following SJNPs treatment (Figure 9g).

We then tested whether NGF administration alone could protect against sepsis‐associated lung injury (Figure 9h). Exogenous NGF significantly prolonged survival in CLP mice, reduced histological damage and inflammatory infiltration, and preserved pulmonary neuronal integrity (Figure 9i,j; Figure S6a). Meanwhile, NGF facilitated the recovery of the tight junction proteins ZO‐1 and Claudin‐1 in septic mice (Figure S6b,c). To place NGF within the SJNPs mechanistic pathway, we depleted macrophages with Lipo, confirming loss of F4/80^+^ cells (Figure S6d). As expected, SJNPs efficacy was lost in macrophage‐depleted mice; however, NGF supplementation partially rescued survival, attenuated lung inflammation, and restored neuronal integrity (Figure 9k,l; Figure S6e), while simultaneously promoting the restoration of the tight junction proteins ZO‐1 and Claudin1, indicating that NGF functions downstream of macrophage modulation(Figure S6f,g).

Collectively, these findings define NGF as a critical effector in the SJNPs‐macrophage‐neuron axis, highlighting neurotrophic factor induction as a mechanistic bridge between immune modulation and neural protection. This work supports macrophage nanotherapy as a rational and translatable strategy for sepsis intervention.

## 3 Discussion

Sepsis remains a formidable clinical challenge, in which organ dysfunction arises from the convergence of uncontrolled inflammation and collateral tissue injury, including damage to peripheral neurons [43, 44]. Although recent immunomodulatory approaches have shown promise, whether macrophages can be therapeutically harnessed to both suppress hyperinflammation and promote neural repair remains to be explored. Here, we identify macrophage–neuron immunometabolic crosstalk as a previously unrecognized therapeutic axis and demonstrate that dual targeting of macrophage polarization and neurotrophic signaling can simultaneously alleviate septic lung injury and preserve neuronal integrity. Our dual‐functional platform, SJNPs, co‐delivers the glutamate production inhibitor JHU083 and the neuroprotective Spm, thereby integrating immunoreprogramming and neuroprotection into therapy. This strategy not only limits inflammatory tissue damage but also activates intrinsic neuronal support mechanisms, offering a systems‐level framework for sepsis intervention.

Lung injury is among the most frequent and severe complications of sepsis, often progressing to acute respiratory distress syndrome (ARDS) [45, 46, 47]. Consistent with clinical and preclinical observations, we observed marked alveolar disruption, inflammatory infiltration, and edema in septic lungs. SJNPs treatment improved histopathological outcomes, reduced immune cell recruitment, and selectively suppressed pro‐inflammatory cytokines, including TNF‐α, IL‐6, IL‐1β, and IFN‐β, that drive the cytokine storm and perpetuate lung damage. These results suggest that SJNPs not only attenuate early‐phase hyperinflammation but also interrupt downstream signaling cascades that perpetuate organ injury.

Central to this therapeutic effect is the phenotypic plasticity of macrophages, whose polarization state dictates the balance between pro‐inflammatory injury (M1) and reparative resolution (M2) [48, 49]. We show that SJNPs shift macrophage polarization toward a reparative M2 phenotype across multiple tissues, including the lung, ileum, and spleen, while suppressing M1‐driven inflammation. Importantly, macrophage depletion abrogated the protective effects of SJNPs, establishing macrophages as indispensable effectors of the therapy. These findings highlight the feasibility of targeting macrophage immunometabolism as a tractable and systemic strategy for restoring immune homeostasis in sepsis.

One of the most unexpected and conceptually significant findings is that reprogramming of macrophages can induce secretion of the neurotrophic factor NGF, leading to both structural preservation and functional protection of pulmonary neurons. While NGF is well recognized in peripheral nerve regeneration, its role in macrophage‐mediated neuroprotection within septic lungs has not been previously defined [50, 51]. We show that M2 macrophage‐derived NGF supports neuronal survival and may dampen neurogenic inflammation through feedback regulation. This is particularly relevant because neuroimmune crosstalk is increasingly recognized as a determinant of local tissue homeostasis. Supplementation of exogenous NGF partially restored therapeutic efficacy in macrophage‐depleted mice, providing direct evidence for NGF's central role in bridging immune modulation and neuroprotection. This study demonstrates that macrophage‐secreted NGF is a key regulator of pulmonary inflammation, offering a unique entry point for dual‐target strategies against septic organ injury.

In summary, this study reframes glutamate metabolism not merely as a metabolic pathway but as an upstream regulator of both immune and neuronal outcomes in sepsis. By embedding metabolic inhibition and neurotrophic enhancement within a single delivery platform, SJNPs represent a rationally designed intervention capable of addressing both immune dysregulation and neurodegeneration. These findings not only expand our mechanistic understanding of macrophage–neuron interactions but also lay a conceptual foundation for precision immunometabolic therapies that simultaneously target multiple organ systems in critical illness.

## 4 Conclusion

Our study identifies macrophage‐neuron immunometabolic crosstalk as a pivotal therapeutic axis in sepsis and establishes dual targeting of macrophage polarization and neurotrophic signaling as a viable strategy to alleviate organ injury. By integrating glutamate metabolic inhibition with neuroprotective support in a single SJNPs platform, we demonstrate simultaneous suppression of hyperinflammation and preservation of pulmonary neuronal integrity. These findings highlight macrophage‐secreted NGF as a central mediator linking immune modulation and neural protection, thereby reframing sepsis therapy at the systems level. Collectively, this work not only advances mechanistic insight into immune‐neural interactions but also provides a conceptual and translational framework for precision interventions that mitigate multiorgan dysfunction in critical illness.

## 5 Experimental Section

### 5.1 Animals

Healthy 7‐8‐week‐old female C57BL/6 mice weighing 21–28 g were used in this study and purchased from Augct Bio, Xi'an. All mice were housed in groups of five per cage under Specific Pathogen Free (SPF) conditions, with a controlled temperature of 21 ± 2 °C, relative humidity of 40%–70%, and a 12 h light/dark cycle. Prior to experimentation, mice were acclimatized to the environment for at least one week.

### 5.2 Cell Lines

RAW264.7 (mouse mononuclear macrophage leukemia cells, Procell, CL‐0190, RRID: CVCL_0493) were purchased on March 1, 2023. PC12 (rat adrenal medullary pheochromocytoma cells, ATCC, CRL‐1721, RRID: CVCL_0481) were purchased on August 7, 2023. They were cultured in Dulbecco's Modified Eagle's Medium (DMEM) supplemented with 10% fetal bovine serum (ExCell Bio, FSP500), penicillin (100 U/mL), and streptomycin (100 µg/mL), respectively, at 37 °C with 5% CO_2_. In this study, the cell lines involved were confirmed to be correct and free of contamination after short tandem repeat (STR) analysis and quality inspection.

### 5.3 Construction and Characterization of JHUNPs

25 mg of JHU083‐PEG was dissolved in 100 µL DMSO, and the DMSO solution was added to 5 mL of ultrapure water and stirred at room temperature for 30 min to obtain JHUNPs. The uniformity of the nanoparticle dispersion was evaluated by the Tyndall effect, and the morphology of the assemblies was observed by transmission electron microscopy, and the nanoparticle particle size was determined by DLS.

### 5.4 Construction and Characterization of SJNPs

25 mg of JHU083‐PEG was dissolved with 40 mg Spm in 100 µL of DMSO, and the DMSO solution was added to 5 mL of ultrapure water and stirred at room temperature for 30 min to obtain SJNPs. The uniformity of the nanoparticle dispersion was evaluated by the Tyndall effect, and the morphology of the assemblies was observed by transmission electron microscopy, and the nanoparticle particle size was determined by DLS.

### 5.5 Stability Monitoring of JHUNPs and SJNPs

To assess the stability of nanoparticles, JHUNPs and SJNPs were dissolved in (Phosphate Buffered Saline) PBS. The particle size as well as the PDI of the nanoparticles were monitored daily by DLS for 7 consecutive days.

### 5.6 Drug Release

100 µL of SJNPs solution was added to the dialysis bag and immersed in 500 mL of PBS at 37 °C with moderate agitation. 1 mL of release liquid was collected at various time intervals, and 1 mL PBS was added to maintain the total volume. We measured drug release from Spm and DON by HPLC. All release tests were performed in triplicate, and average values are reported. The drug loading rate was 33.3%.

### 5.7 BMDM Extraction and Stimulation

BMDMs were isolated from 7‐week‐old female C57BL/6 mice. The isolated cells were cultured in complete DMEM medium containing macrophage colony‐stimulating factor (M‐CSF) (20 ng/mL, Sangon Biotech) at 37 °C and 5% (v/v) CO_2_. After 7 days of culture, adherent cells were considered BMDMs. After subsequent stimulation with DMEM complete medium containing LPS (1 µg/mL) for 6 h, the drugs JHUNPs, Spm, and SJNPs were added (concentrations of JHUNPs 5 µm, Spm 35 µm, respectively).

### 5.8 Flow Cytometry

For cell staining, cells were first washed with PBS and then incubated with antibodies targeting extracellular epitopes of the protein of interest at 4 °C for 1 h, with gentle mixing every 20 min. Next, add the fixative to fix the cells, then use the membrane disruption solution to disrupt the cell membrane. Finally, add the antibody inside the target protein membrane for staining.

For the processing of tissues (such as spleen, lung, and ileum), tissues were first minced into small fragments using scissors, followed by enzymatic digestion with collagenase IV and DNase I at 37 °C for 30 min. After homogenizing the tissue using a 1 mL syringe plunger, rinse the cells on a 70 µm filter with PBS to obtain a single‐cell suspension. Next, the cell suspension is counted to facilitate subsequent antibody staining. The antibody staining process is the same as the cell processing process. Detailed information on all antibodies is provided in the Table S1.

### 5.9 Western Blot

Total protein was extracted from tissues or cells using RIPA lysis buffer supplemented with protease inhibitors and PMSF. Samples were lysed on ice for 40 min, followed by centrifugation at 12 000 rpm for 10 min at 4 °C. The supernatant was collected and denatured with loading buffer. Separate the sample on an SDS‐PAGE gel, then transfer it to a 0.45 µm PVDF membrane. Incubate the membrane in blocking buffer (TBST solution containing 5% non‐fat dry milk) for 1 h. After blocking, incubate the membrane with the primary antibody at 4 °C overnight. The next day, wash the membrane three times with TBST, each time for 8 min. Next, the membrane is incubated with HRP‐labeled secondary antibody at room temperature for 1 h, followed by three washes with TBST, each for 8 min. Finally, the membrane is immersed in ECL buffer, and protein signals are detected using a chemiluminescence imaging system (VILBER‐VILBER FUSION FX6.EDGE). Detailed information on all antibodies is provided in the Table S2.

### 5.10 RNA‐Seq

Total RNA was extracted from samples treated under different conditions using Trizol reagent (ThermoFisher, 15596018). The RNA was then purified and measured using a Bioanalyzer 2100 and RNA 6000 Nano LabChip Kit (Agilent, CA, USA, 5067‐1511). Only samples with an RNA integrity number (RIN) >7.0 were used for library construction. RNA sequencing (RNA‐seq) was performed on the Illumina X10 platform by LC Sciences (Hangzhou, Zhejiang, China). Genes with a false discovery rate (FDR) <0.05 and an absolute fold change ≥2 were considered differentially expressed. Subsequent analyses were conducted based on these differentially expressed genes.

### 5.11 Sepsis Model

CLP‐induced sepsis model: Prior to surgery, the abdominal hair of C57BL/6 mice was shaved. Mice were anesthetized with isoflurane at the beginning of the procedure. A midline abdominal incision was made to expose the cecum, which was ligated at approximately 35% of the distance between the distal end and the base using a 5‐0 suture. Following ligation, a 21‐gauge needle was used to puncture the cecum, and a drop of feces was expelled. The incision was then closed with sutures in layers. Postoperatively, the mice were administered saline for fluid resuscitation. For the sham surgery group, only an exploratory laparotomy was performed, with the cecum removed from the abdominal cavity without ligation or puncture. The drugs (JHUNPs 0.5 mg/kg, Spm 1 mg/kg, SJNPs 1.3 mg/kg) were administered through the tail vein 1 h after surgery. Mortality of the mice was monitored over 72 h. (b) LPS‐induced sepsis model: LPS (25 mg/kg) was injected into the abdominal cavity of C57BL/6 mice, and observations were made for 72 h.

### 5.12 Collect BALF

After euthanizing C57BL/6 mice, the limbs were immobilized, and the neck surface was disinfected with 75% ethanol. The trachea was carefully exposed by dissecting the surrounding muscles and adipose tissue using scissors and forceps. A small triangular incision was made above the trachea, and a gavage needle was inserted and secured with suture thread to prevent dislodgement. A 1 mL syringe was used to slowly instill 500 µL of PBS into the trachea while gently massaging the lungs. After 20 s, the fluid was aspirated. This lavage procedure was typically repeated three times.

### 5.13 H&E Staining

Mouse organs were harvested and fixed in 4% paraformaldehyde (PFA; Servicebio, G1101). After gradient dehydration and paraffin embedding, 4 µm sections were made with a slicer. The sections were stained with hematoxylin for 10 min and washed with running water. Hematoxylin stained the nuclei and ribosomes a blue‐purple color. Excess nuclear and cytoplasmic stain was removed with acid alcohol, followed by washing with double‐distilled water. The sections were then counterstained with eosin for 2 min to stain the cytoplasm and extracellular matrix components pink‐red. Subsequently, the slides were dehydrated through graded ethanol solutions (95% and absolute ethanol), cleared, and mounted with neutral resin (Solarbio, G8590). After natural drying, images were captured under a light microscope.

### 5.14 IF Staining

Cell coverslips and tissue sections were stained for immunofluorescence and visualized using confocal microscopy. Membrane proteins were incubated with primary antibody at 4 °C for 12 h. As for intracellular proteins, they need to be permeabilized with PBS solution containing 0.1% Triton for 10 min before incubation with the primary antibody. Then, the appropriate secondary antibody is selected for incubation. Finally, the nuclei were counterstained with DAPI.

### 5.15 Preparation of RAW264.7 Conditioned Medium

RAW264.7 cells were seeded in six‐well plates and allowed to adhere for 12 h. The culture medium was then replaced with complete DMEM containing 1 µg/mL LPS, and cells were incubated for 6 h. Subsequently, cells were treated with the following compounds according to group assignment: JHUNPs, Spm, and SJNPs. After 24 h of drug treatment, the supernatant was discarded, and fresh DMEM complete medium was added to continue incubation for 24 h. Finally, the supernatant was collected and centrifuged using 3000 rpm for 10 min, and the resulting supernatant was the conditioned medium.

### 5.16 Apoptosis

To determine the apoptosis of PC12 cells after treatment with inflammatory factors. PC12 cells were inoculated in six‐well plates and cultured until the cell density reached 70%. Subsequently, different conditioned media of RAW264.7 were added and incubation was continued for 24 h. All cells were collected and stained using Annexin V/PI Apoptosis Kit (Dojindo Laboratories, AD10) according to the manufacturer's instructions. Data were analyzed using a BDFACSelesta flow cytometer, and flow cytometry results were processed using FlowJo X software.

### 5.17 Direct Contact Assay

CFSE‐labeled PC12 cells were co‐cultured with RAW264.7 cells. First, a certain number of PC12 cells were added to 1 mL of DMEM incomplete medium with 1 µL of 5 µm CFSE fluorescent dye and incubated at 37 °C for 20 min. After incubation, the cells were washed three times using PBS and then added to a six‐well plate cultured with RAW264.7 cells. After 12 h, the medium was replaced with complete DMEM medium containing 1 µg/mL LPS, and the incubation was continued for 24 h. At the end of culture, the number of PC12 cells was detected using flow cytometry and immunofluorescence.

### 5.18 Neurotrophic Factor Assay

Following LPS stimulation of RAW264.7 cells and 20 h of drug treatment, BFA was added to the culture medium and incubated for an additional 5 h to block protein secretion. Subsequently, total protein was extracted from the cells, and Western blot analysis was performed to detect several major classes of neurotrophic factors. Detailed information on all antibodies is provided in the Table S2.

### 5.19 Elisa

The cell culture medium or BALF was centrifuged at 3000 rpm for 10 min, and the supernatant was collected for analysis. Serum was collected from coagulated blood by centrifugation (3000 rpm) twice at 4 °C. All samples were assayed according to the manufacturer's instructions. The ELISA kits used in this study are listed in Table S3.

### 5.20 Statistical Analysis

The data were presented as means SD. Two‐sided unpaired t‐tests were used to compare statistical differences between groups. One‐way ANOVA with Tukey's multiple comparisons was used to examine statistical differences for studies of multiple samples. The statistical analysis program GraphPad Prism version 9 was used for all calculations. The statistical significance was determined using a cutoff of *p*<0.05. Significant levels were denoted by the following symbols: ^*^ *p*<0.05, ^**^ *p*<0.01, ^***^ *p*<0.001, and ^****^ *p*<0.0001.

## Author Contributions

W. W., Y. H., X. L., and Y C. carried out the majority of experiments. B. Z., R. F., J. D., J. Y., and S. F. technically supported and revised the manuscript. W. Z., J. S., and W. G. designed and supervised the study. All authors contributed to the data analysis. W. W. wrote the manuscript. All authors reviewed and gave final approval of the manuscript.

## Funding

This work was supported by the National Natural Science Foundation of China (22577101, 82374074, 82173682), the Shenzhen Science and Technology Program (JCYJ20250604183359078), the CAMS Innovation Fund for Medical Sciences (2022‐I2M‐1‐015), and the Innovation Foundation for Doctor Dissertation of Northwestern Polytechnical University (ZX2025005).

## Ethics Statement

All animal experiments were approved by and conducted in accordance with the guidelines of the Institutional Animal Care and Use Committee of Northwestern Polytechnical University. All the experiments involving animals were performed according to protocols approved by the Institutional Animal Care and Use Committee of Northwestern Polytechnical University (202401210).

## Conflicts of Interest

The authors declare no conflicts of interest.

## Supporting information




**Supporting File**: advs74463‐sup‐0001‐SuppMat.docx.

## Data Availability

Raw data for the bulk RNA‐seq analysis comparing JHU083 and control BMDM cells have been deposited in the GEO database under accession number GSE296887. Raw data for the bulk RNA‐seq analysis comparing CLP and control PBMC cells have been deposited in the GEO database under accession number GSE296770.
