# Supplementary material for: Nanotherapeutic Macrophage‐Neuro Reprogramming Through Immunometabolic Crosstalk Mitigates Sepsis‐Induced Lung Injury and Neurologic Damage
Source: Adv Sci (Weinh). 2026 Feb 16;13(24):e20665. doi: 10.1002/advs.202520665 (PMC13116209; doi:10.1002/advs.202520665)
Supplement: Supplementary file 1 — Supporting File: advs74463‐sup‐0001‐SuppMat.docx. [file ADVS-13-e20665-s001.docx]

**Supporting information**

**Nanotherapeutic macrophage-neuro reprogramming through immunometabolic crosstalk mitigates sepsis-induced lung injury and neurologic damage**

*Wenhui Wang^#^, Yongrui Hai^#^, Xintong Lu^#^, Ye Chen^#^, Bingjie Zhang, Renming Fan, Jiarui Dou, Jiaxin Yan, Shuo Fu, Wen Zhang^*^, Junke Song^*^, Gaofei Wei^*^*

#These authors contributed equally.

*Corresponding author. Email: zhangwen9010@imm.ac.cn (W.Z.); smilejunke@imm.ac.cn (J.S.); weigf0605@163.com (G.W.)

**This PDF file includes:**

Supplementary Text

Scheme S1

Tables S1 to S3

Figures S1 to S6

Supplementary Text

**Scheme S1. Synthesis of JHU083-PEG.** Reaction and conditions: (a)m-PEG7-CH2COOH, HOAT, DIPEA, DCM, DMF, 0℃ to RT; (b)TFA, L-Norleucine, 6-diazo-5-oxo-, ethyl ester, EDCI, DCM, 0℃ to RT.

**Synthesis of CY122 (a)：** m-PEG₇-CH₂COOH (100 mg, 0.24 mmol) and N-hydroxy-7-azabenzotriazole (50 mg, 0.36 mmol) were dissolved in dry DCM (5 mL), and 10% DMF (v/v) was added as a cosolvent. The solution was cooled to 0°C, followed by the addition of N,N′-dicyclohexylcarbodiimide (74 mg, 0.36 mmol). After stirring for 15 min, L-leucine tert-butyl ester hydrochloride (84 mg, 0.36 mmol) and DIPEA (93 mg, 0.72 mmol) were added sequentially. The reaction was carried out at room temperature for 12 h. TLC monitoring showed that the starting material was completely converted. The reaction was quenched with saturated NH₄Cl solution (10 mL), and extracted with DCM (3 × 15 mL). The combined organic phases were dried over anhydrous Na₂SO₄, filtered, and concentrated. The crude product was purified by silica gel column chromatography (gradient elution with DCM/MeOH) to obtain the target compound as a colorless oil (74 mg, 0.127 mmol, yield 52.9%). ^1^H NMR (500 MHz, CDCl_3_) δ 6.65 (d, *J* = 8.1 Hz, 1H), 4.47 (td, *J* = 8.5, 5.5 Hz, 1H), 3.71 (d, *J* = 5.8 Hz, 2H), 3.63 (d, *J* = 5.2 Hz, 26H), 3.55 – 3.50 (m, 2H), 3.36 (s, 3H), 2.48 (t, *J* = 5.8 Hz, 2H), 1.70 – 1.54 (m, 2H), 1.51 – 1.45 (m, 1H), 1.43 (s, 9H), 0.92 (dd, *J* = 6.5, 2.7 Hz, 6H).^13^C NMR (126 MHz, CDCl_3_) δ173.14, 171.20, 82.83, 72.02, 70.65(11), 70.50, 70.45, 67.33, 59.11, 51.34, 41.88, 37.00, 28.10(3), 25.02, 22.92, 22.25. MS (ESI): *m/z* [M + Na]^+^604.70.

**Synthesis of JHU083-PEG：** CY122 (100 mg, 0.172 mmol) was dissolved in anhydrous DCM (5 mL), cooled to 0℃ in an ice bath, and trifluoroacetic acid (196 mg, 1.72 mmol) was added dropwise slowly. The reaction system was warmed to room temperature and stirring was continued for 2 h. TLC showed the complete disappearance of the starting material. The reaction solution was concentrated under reduced pressure to obtain the free acid intermediate. The above free acid and 1-(3-dimethylaminopropyl)-3-ethylcarbodiimide hydrochloride (50 mg, 0.258 mmol) were dissolved in anhydrous DCM (5 mL), cooled to 0℃, then ethyl 6-diazo-5-oxo-L-norleucinate (35 mg, 0.172 mmol) was added, and the reaction was carried out at room temperature for 16 h. After TLC monitoring indicated the reaction was complete, the reaction mixture was quenched with saturated NH₄Cl solution (10 mL) and extracted with DCM (3 × 15 mL). The combined organic phases were dried over anhydrous Na₂SO₄, filtered, and concentrated under reduced pressure. The crude product was purified by silica gel column chromatography (gradient elution with DCM/MeOH) to afford the target product as a yellow oil (70 mg, 0.099 mmol, yield 57.6%). ^1^H NMR (500 MHz, CDCl3) δ 7.00 (d, *J* = 7.8 Hz, 1H), 6.89 (s, 1H), 5.54 (s, 1H), 4.45 (td, *J* = 8.3, 4.4 Hz, 1H), 4.35 (ddd, *J* = 9.1, 7.4, 4.9 Hz, 1H), 4.14 (qd, *J* = 7.1, 2.8 Hz, 2H), 3.77 (t, *J* = 5.6 Hz, 2H), 3.74 – 3.59 (m, 26H), 3.55 (dd, *J* = 5.9, 3.2 Hz, 2H), 3.37 (s, 3H), 2.67 (dt, *J* = 10.9, 5.6 Hz, 1H), 2.58 (dt, *J* = 15.1, 5.5 Hz, 1H), 2.41 (s, 2H), 2.19 (ddt, *J* = 12.6, 7.9, 4.8 Hz, 1H), 2.06 – 1.93 (m, 1H), 1.69 – 1.62 (m, 2H), 1.25 (t, *J* = 7.1 Hz, 4H), 0.98 – 0.87 (m, 6H).^13^C NMR (126 MHz, CDCl3) δ 193.82, 172.21, 171.54, 171.27, 71.65, 70.30 (11), 70.09, 70.01, 66.94, 61.17, 58.72, 54.60, 53.40, 51.57, 40.67, 36.50, 36.19, 24.45, 22.72, 21.75 (2), 13.88. MS (ESI): *m/z* [M + Na]^+^729.66.


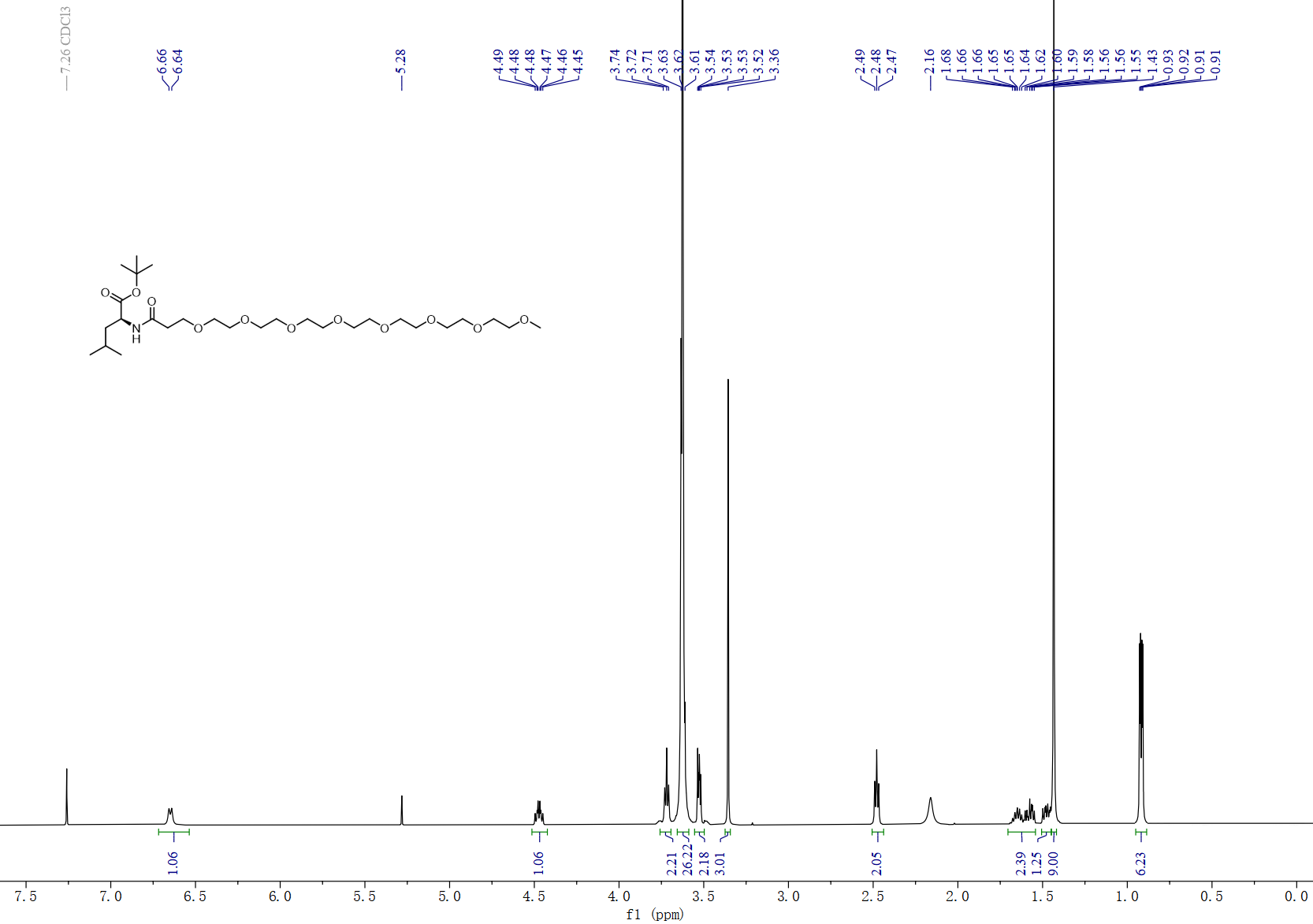


^1^H-NMR specture of CY122


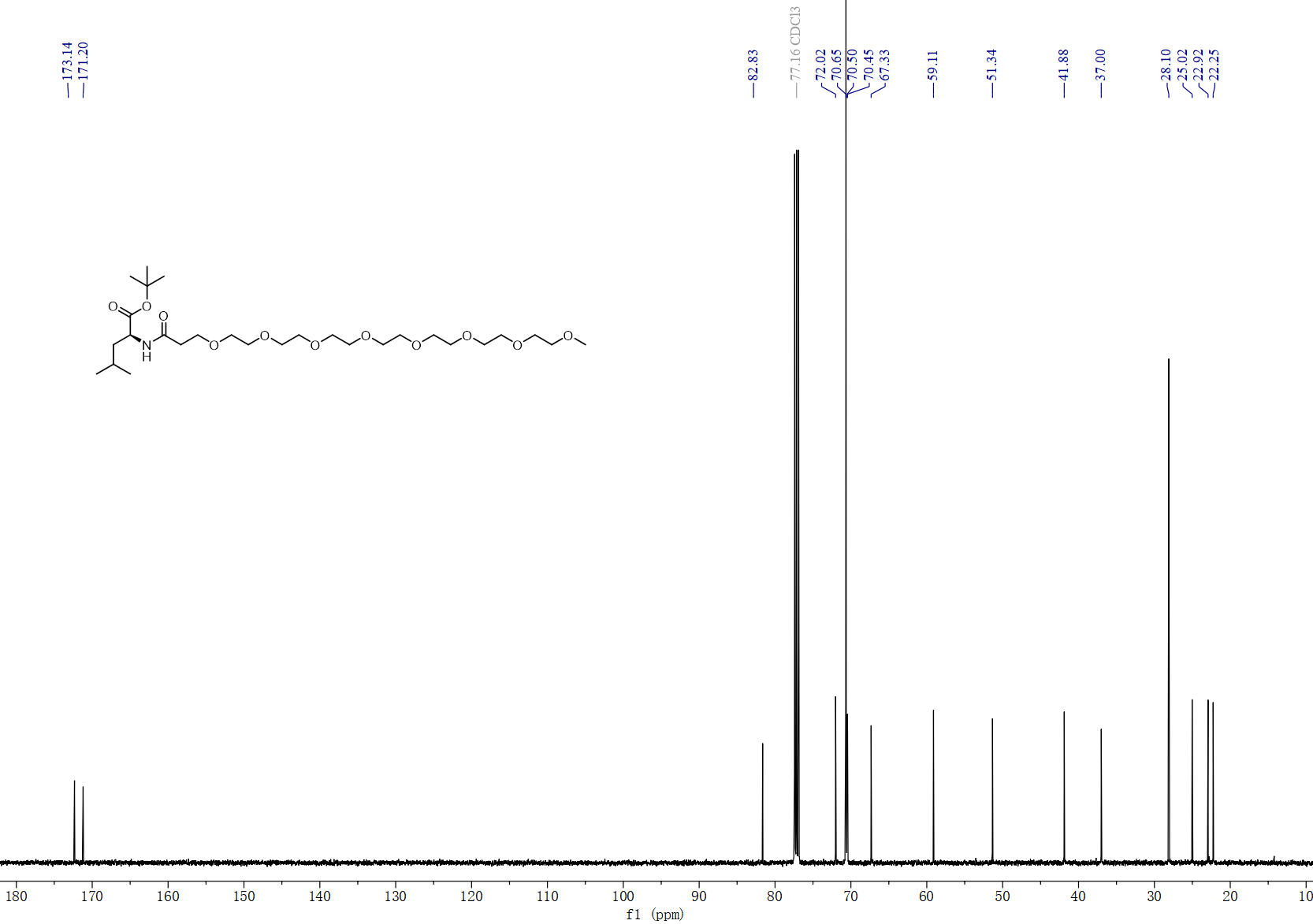


^13^C-NMR specture of CY122

MS Spectrum of CY122


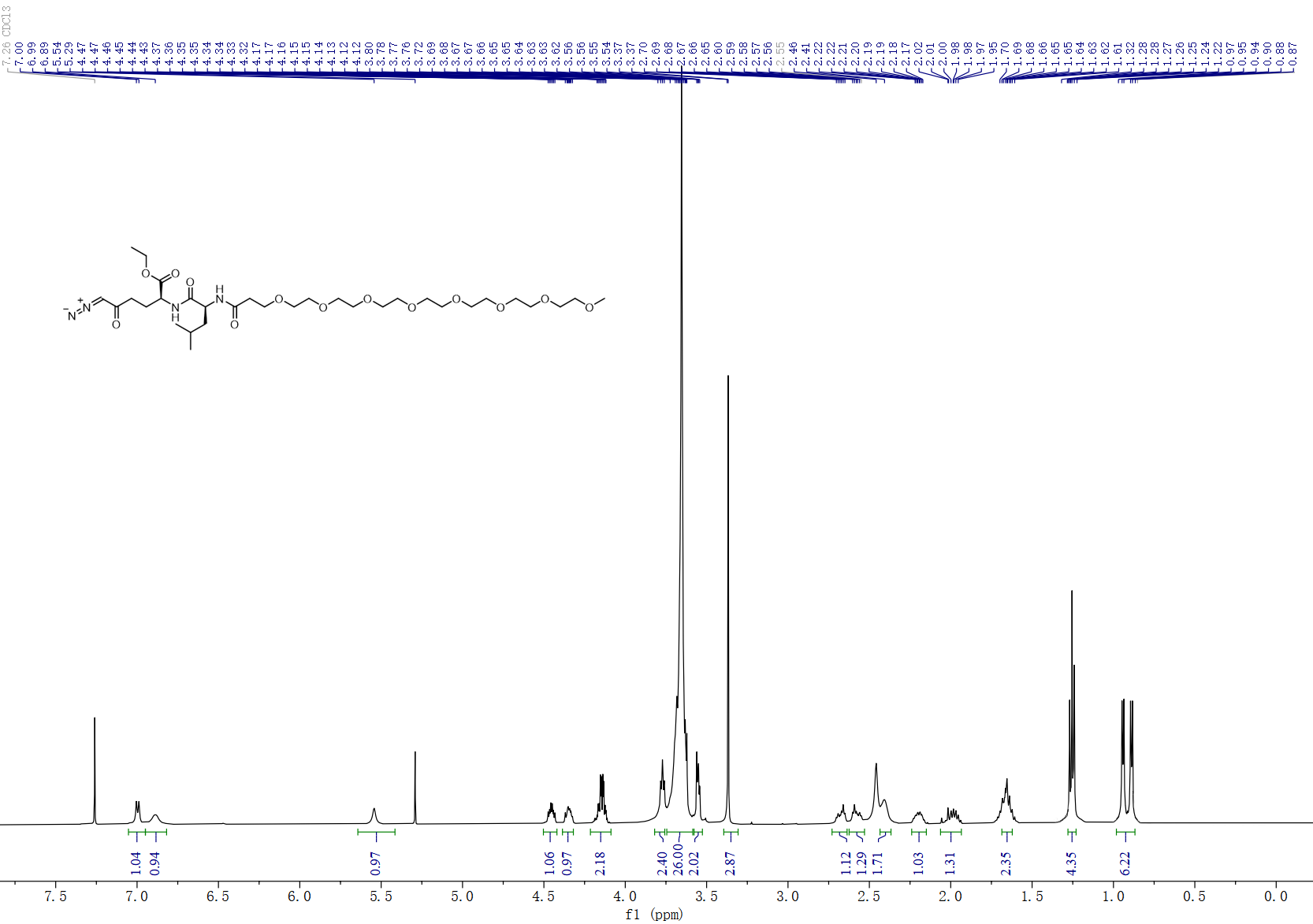


^1^H-NMR specture of JHU083-PEG


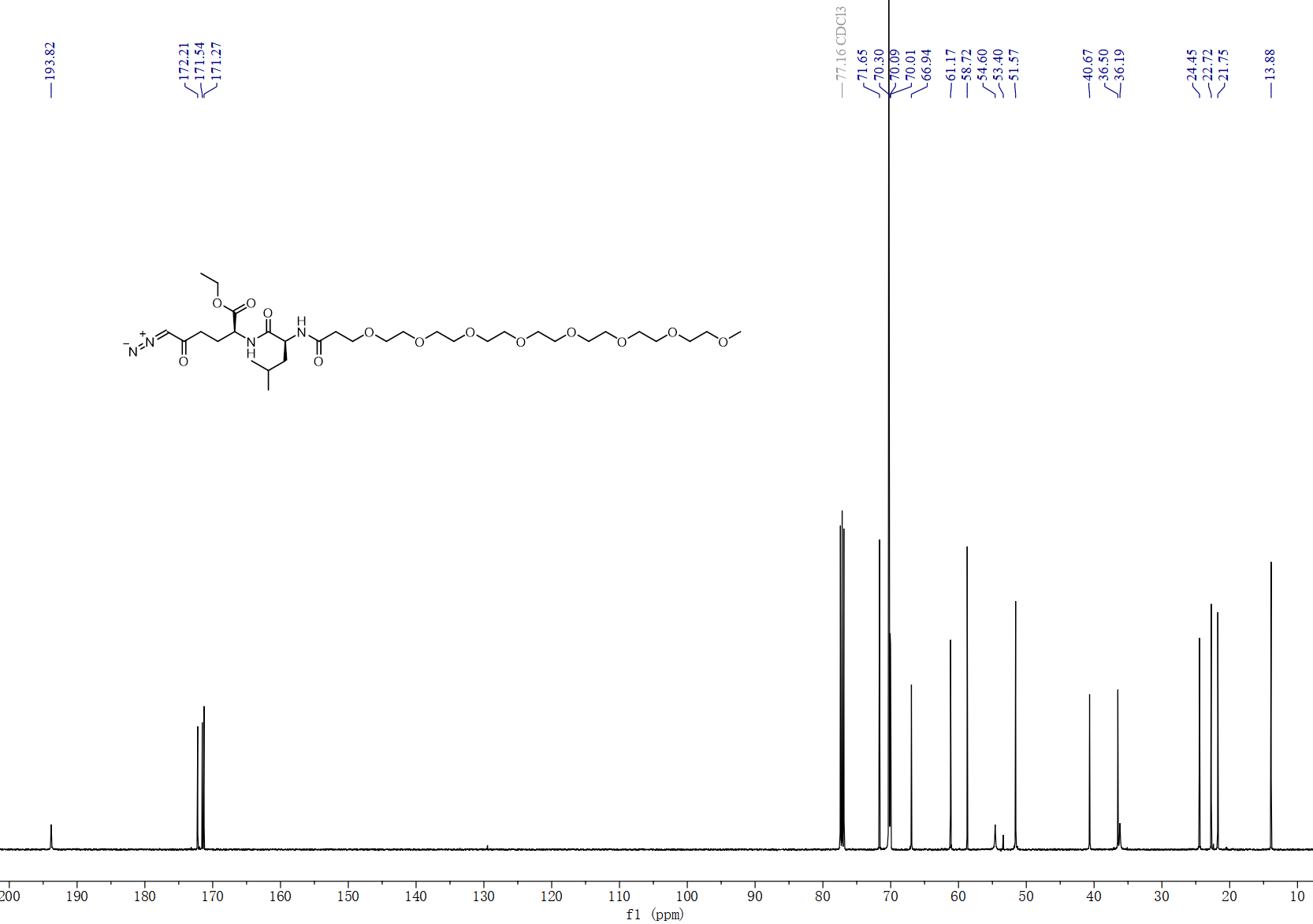


^13^C-NMR specture of JHU083-PEG

MS Spectrum of JHU083-PEG

**Supplementary Table S1. Antibody used for cytometry.**

| Antibody | Dilution | Catalog | Manufacturer |
| --- | --- | --- | --- |
| FITC anti-mouse F4/80 Antibody | 1:200 | 123108  RRID: AB_893502 | Biolegend |
| PerCP-Cy5.5 rat anti-mouse CD11b | 1:80 | 85601  RRID: AB_3717388 | CST |
| APC rat anti-mouse CD86 | 1:300 | 84393  RRID: AB_3665017 | CST |
| PE anti-mouse CD206 Antibody | 1:40 | 141706  RRID: AB_10895754 | Biolegend |

**Supplementary Table S2. Antibody used for IF and Western blot.**

| Antibody | Catalog | Manufacturer |
| --- | --- | --- |
| Anti-Vinculin antibody | ab129002  RRID: AB_11144129 | Abcam |
| Anti-beta Actin antibody | ab8226  RRID: AB_306371 | Abcam |
| Anti-GAPDH antibody | ab181602  RRID: AB_2630358 | Abcam |
| CD206/MRC1(E6T5J)XP® Rabbit mAb | 24595  RRID: AB_2892682 | CST |
| CD86 | 19589  RRID: AB_2892094 | CST |
| iNOS Rabbit antibody | ab178945  RRID: AB_2861417 | Abcam |
| CD86 Rat antibody | ab238468  RRID: AB_3717389 | Abcam |
| F4/80 (D4C8V) XP® Rabbit mAb (AlexaFluor®488) | 27076  RRID: AB_3717387 | CST |
| Antibody | Catalog | Manufacturer |
| Goat Anti-mouse IgG H&L | ab6708  RRID: AB_956005 | Abcam |
| Goat Anti-Rabbit IgG H&L | ab205718  RRID: AB_2819160 | Abcam |
| Donkey Anti-Rat IgG H&L | SA00001-10  RRID: AB_2935610 | Proteintech |
| Goat Anti-Rabbit IgG H&L (AlexaFluor®647) | ab150079  RRID: AB_2722623 | Abcam |
| Goat Anti-Rabbit IgG H&L(AlexaFluor®488) | ab150077  RRID: AB_2630356 | Abcam |
| Goat Anti-Rat IgG H&L (Cy3®) | GB21302  RRID: AB_2936331 | Servicebio |
| Anti-NeuN antibody | 177487  RRID: AB_2532109 | Abcam |
| ZO-1 Polyclonal antibody | 21773-1-AP  RRID: AB_10733242 | Proteintech |
| Anti-Claudin1 antibody | ab307692  RRID: AB_3083082 | Abcam |
| GDNF | 26179-1-AP  RRID: AB_3085846 | Proteintech |
| NGF | sc-32300  RRID: AB_628020 | Santa Cruz Biotechnology |
| NT4 | ab150437  RRID: AB_3717386 | Abcam |

**Supplementary Table S3. ELISA assay kits.**

| Antibody | Catalog | Manufacturer |
| --- | --- | --- |
| The Mouse TNF-α ELISA Kit | 88-7324-88  RRID: AB_2575080 | Thermo Fisher |
| The Mouse IL-6 ELISA Kit | 88-7064-88  RRID: AB_2574990 | Thermo Fisher |
| The Mouse IL-1β ELISA Kit | 88-7013-22  RRID: AB_2574942 | Thermo Fisher |
| The Mouse IFN-β ELISA Kit | 42400-1  RRID: AB_3717390 | Thermo Fisher |


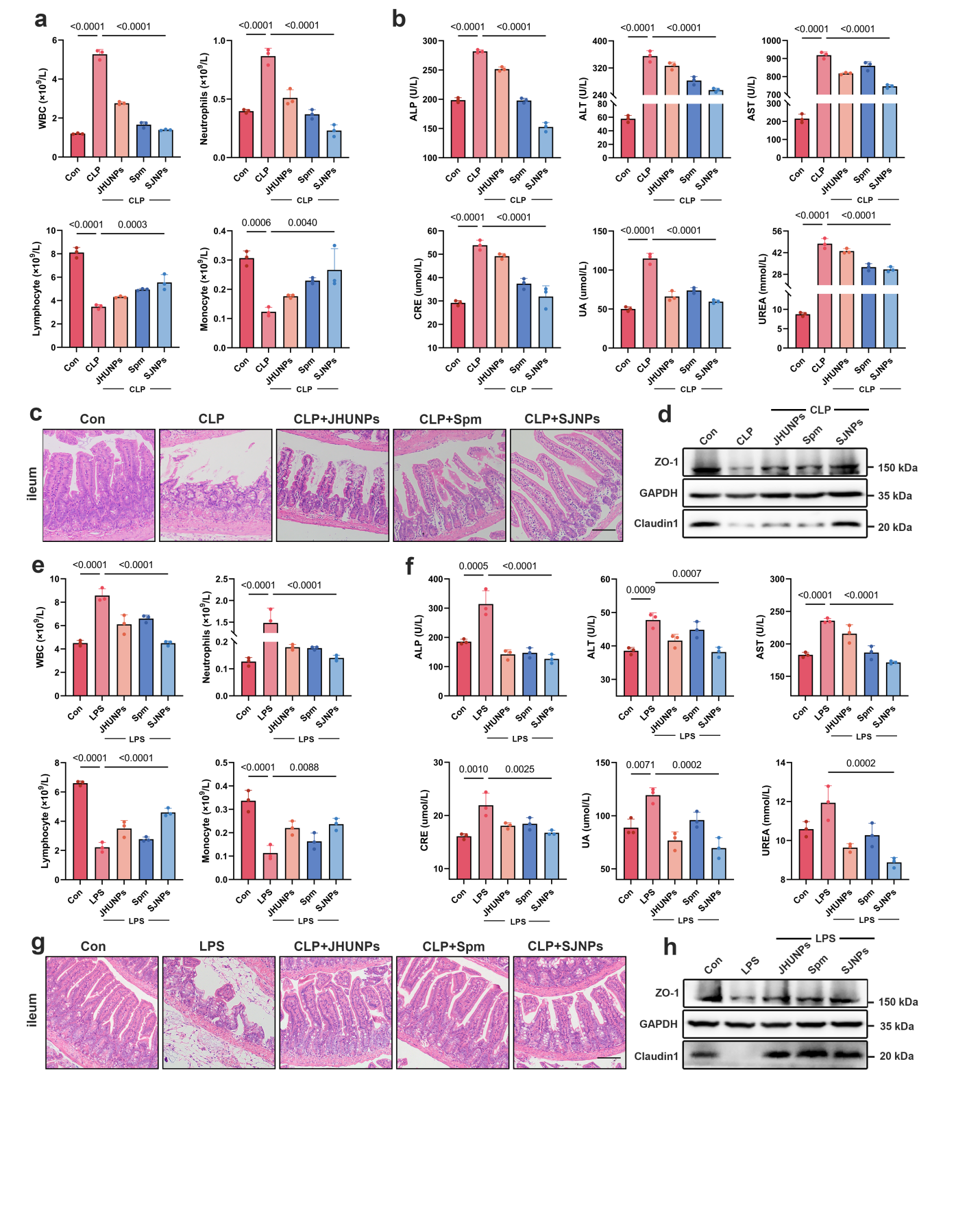


**Figure S1. SJNPs normalize physiological indicators in sepsis models.** (a) Routine blood test results in the CLP model. (b) Liver function indicators (ALP, ALT, AST) and kidney function indicators (CRE, UA, UREA) in the CLP model (n = 3 per group). (c) Representative H&E staining of ileum tissue from CLP-induced septic mice. Scale bar, 100 μm. (d) WB analysis of ZO-1 and Claudin1 expression in ileum tissue from the CLP model. (e) Routine blood test results in the LPS model. (f) Liver (ALP, ALT, AST) and kidney (CRE, UA, UREA) function indicators in the LPS model (n = 3 per group). (g) Representative H&E staining of ileum tissue from LPS-induced septic mice. Scale bar, 100 μm. (h) WB analysis of ZO-1 and Claudin1 expression in ileum tissue from the LPS model. Error bars represent means ± SD. Diﬀerences between groups were tested using one-way ANOVA followed by Tukey’s multiple comparisons test, or unpaired Student’s t-test.

**
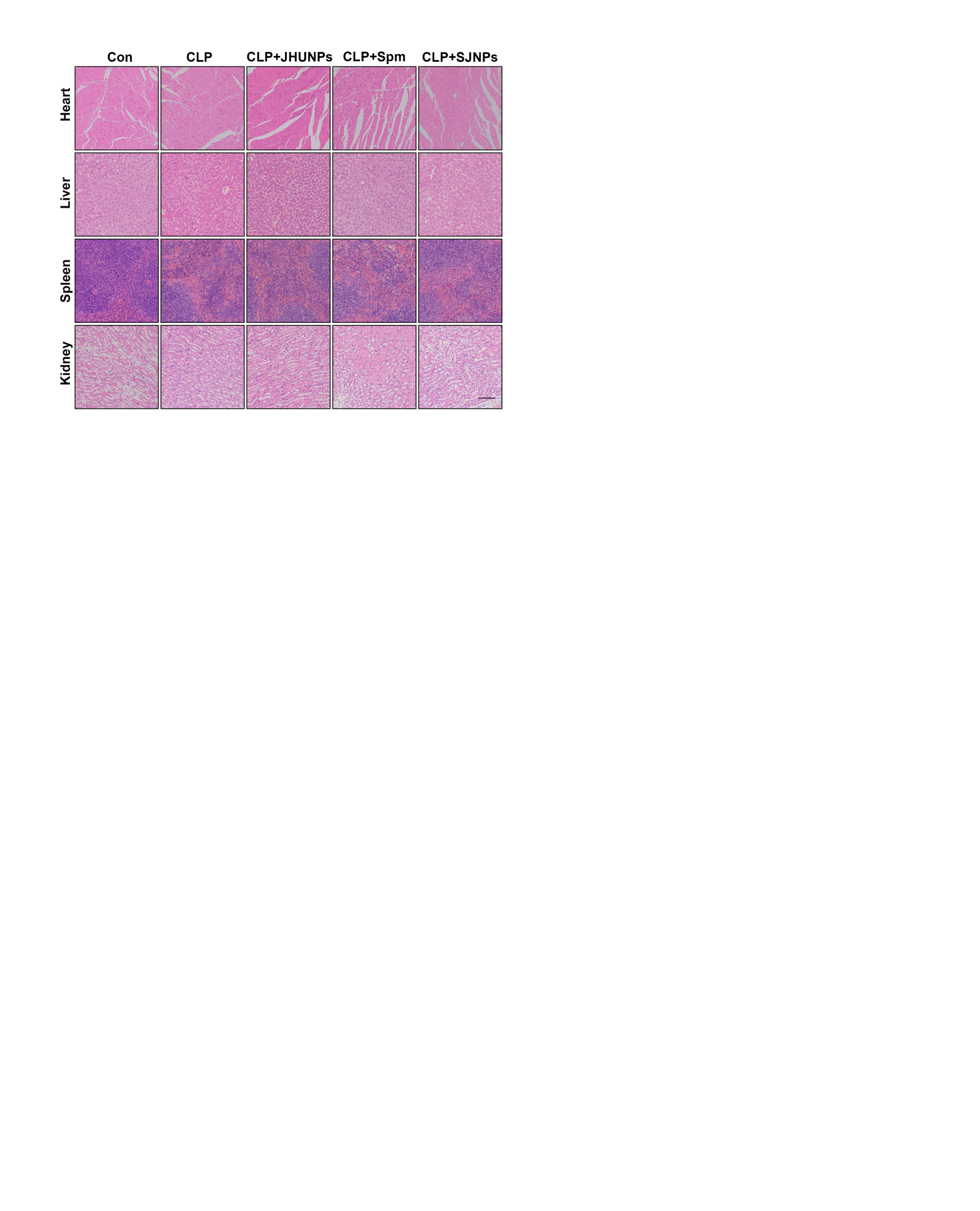
**

**Figure S2. *In vivo* safety assessment.** Histological examination by H&E staining of the heart, liver, spleen, and kidney tissues.


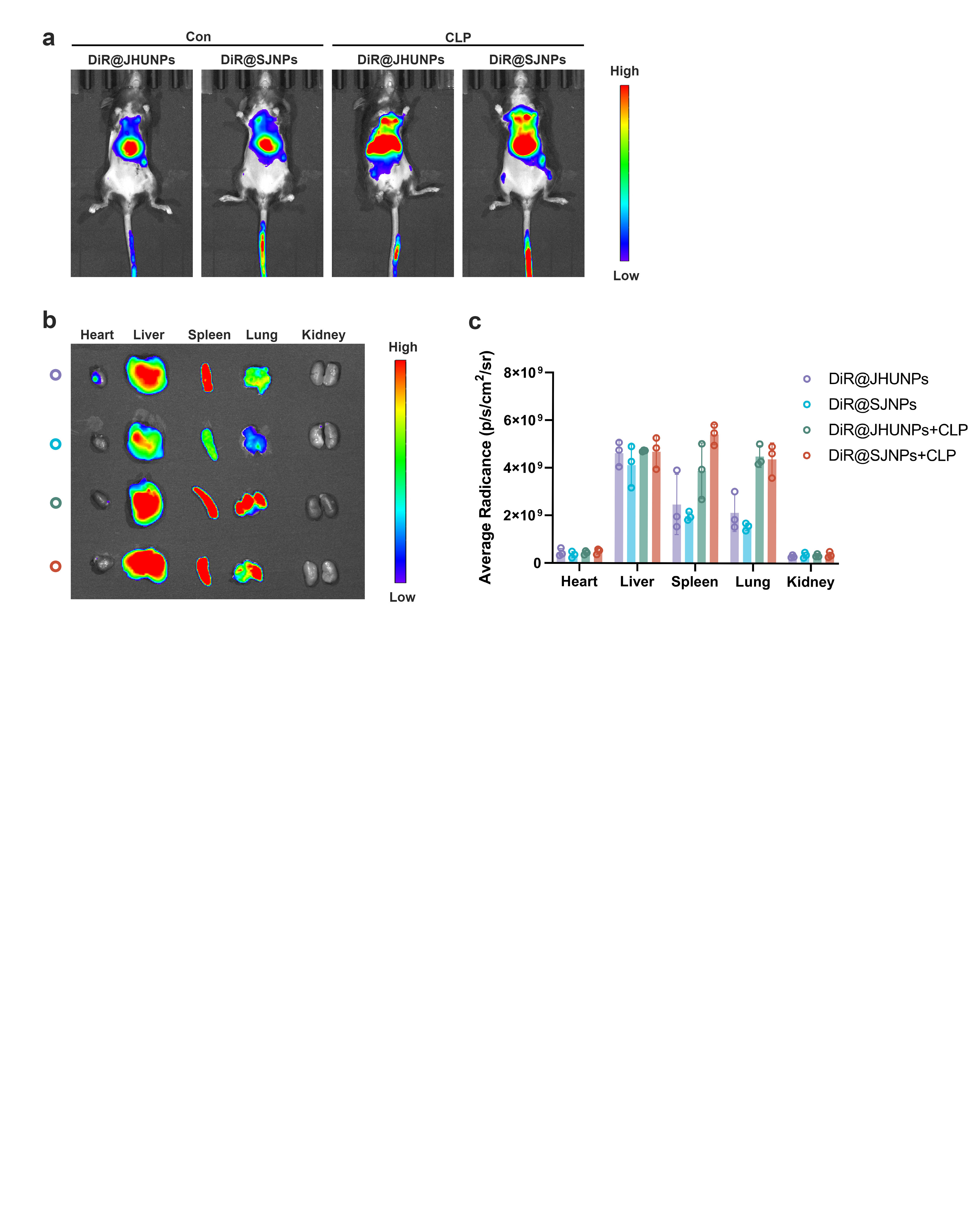


**Figure S3. SJNPs preferentially accumulated in the lung tissue of septic mice.** (a) *In vivo* fluorescence imaging following tail vein injection of JHUNPs and SJNPs in normal and septic mice. (b) Fluorescence imaging of the major organs isolated from mice. (c) Quantitative fluorescence intensity of major organs in vivo (n = 3 per group). Error bars represent means ± SD. Diﬀerences between groups were tested using one-way ANOVA followed by Tukey’s multiple comparisons test, or unpaired Student’s t-test.


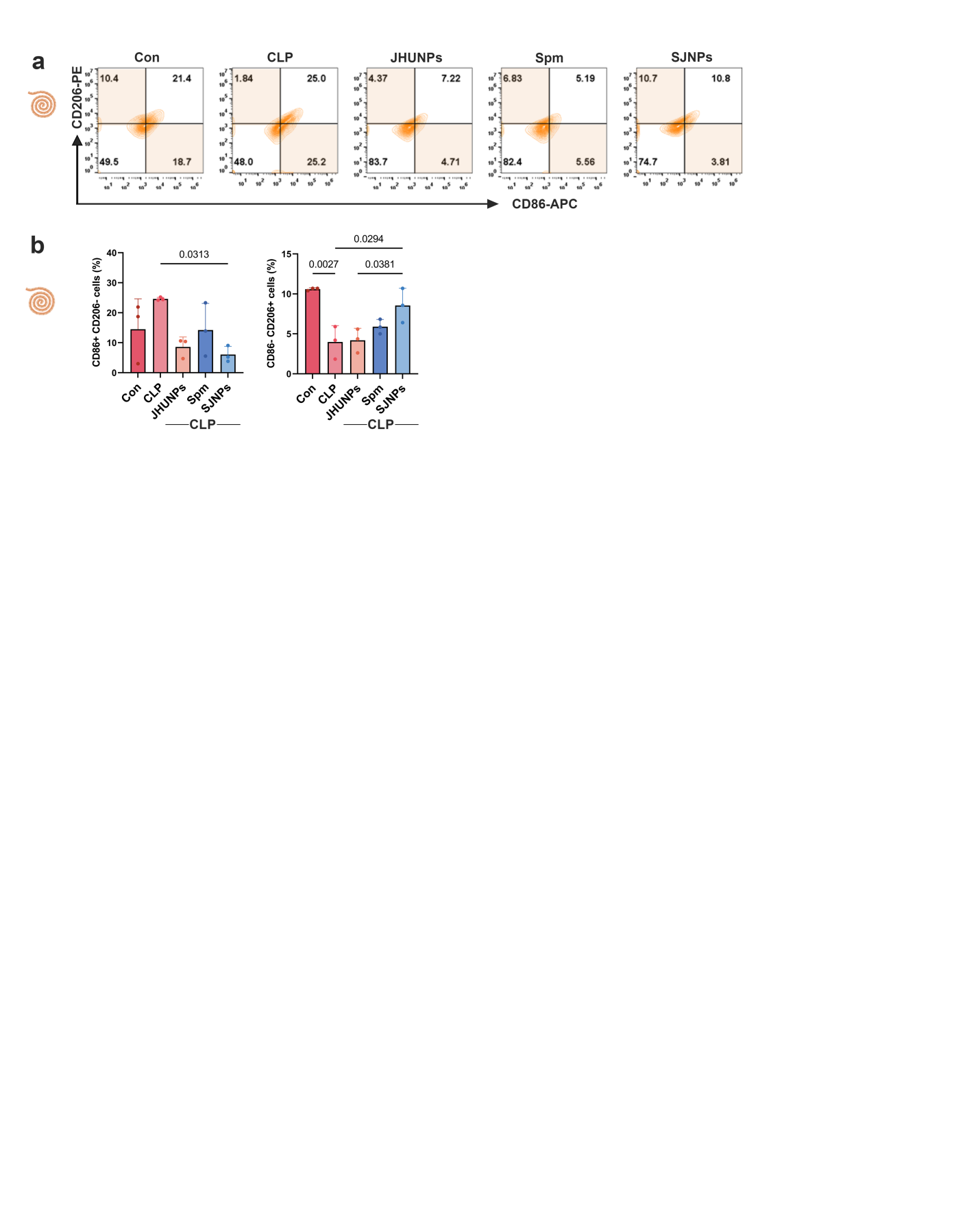


Figure S4. Phenotypic changes in ileum tissue macrophages detected by flow cytometry. (a) Flow cytometric analysis of CD86 and CD206 expression in ileum, from septic mice with or without SJNPs treatment. (b) Quantification of CD86, CD206 expression level. Error bars represent means ± SD. Diﬀerences between groups were tested using one-way ANOVA followed by Tukey’s multiple comparisons test, or unpaired Student’s t-test. Error bars represent means ± SD. Diﬀerences between groups were tested using one-way ANOVA followed by Tukey’s multiple comparisons test, or unpaired Student’s t-test.


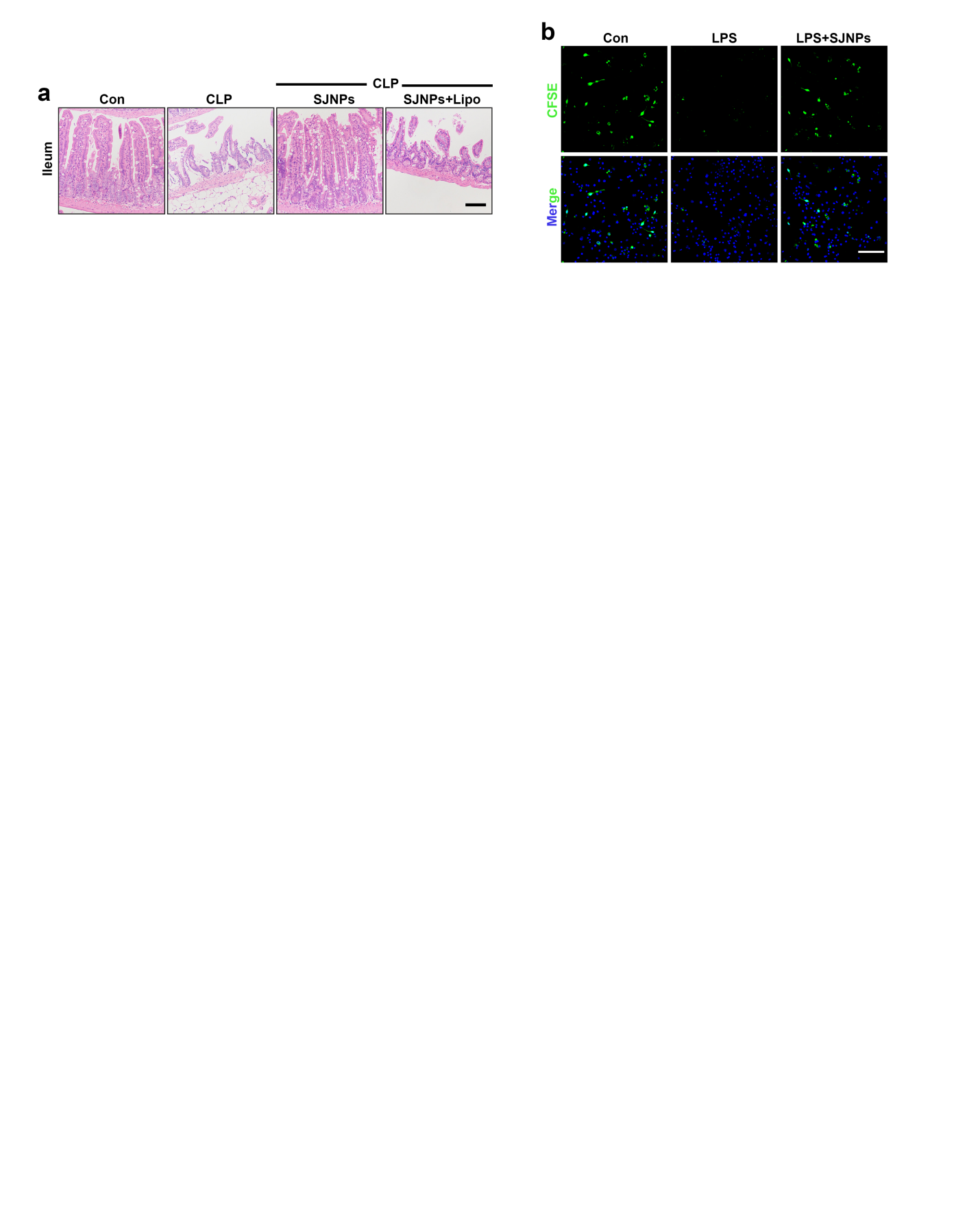


Figure S5. Macrophages are critical for sepsis treatment. (a) Histological examination of ileum tissue by H&E staining in the CLP model following macrophage depletion. Scale bar, 100 μm. (b) RAW264.7 cells were co-cultured with CFSE-labeled PC12 cells for 12 h, followed by treatment with LPS and SJNPs. The number of PC12 cells was then assessed by immunofluorescence after 24 h of culture. Scale bar, 100 μm.


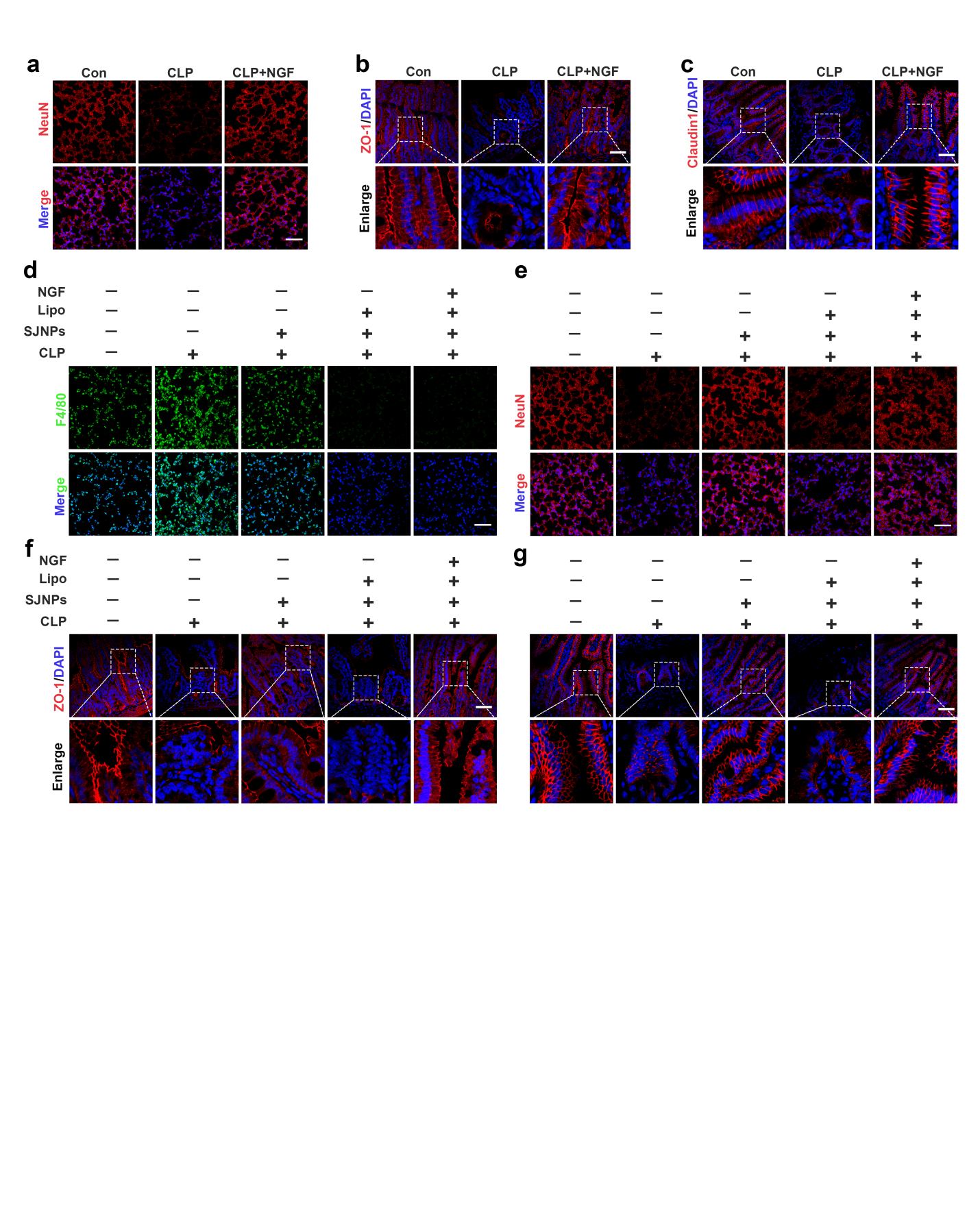


Figure S6. Neural expression in lung tissue. (a) The effect of NGF on lung tissue nerves in the CLP model, labeled with the neuronal marker NeuN. Scale bar, 50 μm. (b, c) Representative immunofluorescence images of ileum sections stained for tight junction proteins ZO-1 and Claudin1 in the CLP model. Scale bars, 100 μm. (d) Immunofluorescence staining of the macrophage marker F4/80 in lung tissue. Scale bar, 50 μm. (e) Detection of NeuN levels in lung tissue from the CLP model by immunofluorescence. Scale bar, 50 μm. (f, g) Representative immunofluorescence images of ileum sections stained for tight junction proteins ZO-1 and Claudin1 in the CLP model. Scale bars, 100 μm.
